# Towards rainbow portable Cytophone with laser diodes for global disease diagnostics

**DOI:** 10.1038/s41598-022-11452-w

**Published:** 2022-05-23

**Authors:** Hind J. Jawad, Aayire C. Yadem, Yulian A. Menyaev, Mustafa Sarimollaoglu, Jillian N. Armstrong, Fumiya Watanabe, Alexandru S. Biris, Jason S. Stumhofer, Dmitry Nedosekin, James Y. Suen, Sunil Parikh, Vladimir P. Zharov

**Affiliations:** 1 Center for Integrative Nanotechnology Sciences, University of Arkansas at Little Rock, 2801 S. University Ave., Little Rock, AR 72204 USA; 2 Arkansas Nanomedicine Center, University of Arkansas for Medical Sciences, 4301 W. Markham St., Little Rock, AR 72205 USA; 3 Department of Physics, University of Baghdad, Al-Jadriya, Baghdad 10071 Iraq; 4 Yale School of Public Health, 60 College St, New Haven, CT 06520 USA; 5 Department of Microbiology and Immunology, University of Arkansas for Medical Sciences, 4301 W. Markham St., Little Rock, AR 72205 USA; 6 College of Medicine, University of Arkansas for Medical Sciences, Little Rock, AR 72205 USA; 7 Department of Otolaryngology, College of Medicine, University of Arkansas for Medical Sciences, Little Rock, AR 72205 USA; 8 CytoAstra, LLC, 401 South Cedar St., Little Rock, AR 72205 USA

**Keywords:** Flow cytometry, Malaria

## Abstract

In vivo, Cytophone has demonstrated the capability for the early diagnosis of cancer, infection, and cardiovascular disorders through photoacoustic detection of circulating disease markers directly in the bloodstream with an unprecedented 1,000-fold improvement in sensitivity. Nevertheless, a Cytophone with higher specificity and portability is urgently needed. Here, we introduce a novel Cytophone platform that integrates a miniature multispectral laser diode array, time-color coding, and high-speed time-resolved signal processing. Using two-color (808 nm/915 nm) laser diodes, we demonstrated spectral identification of white and red clots, melanoma cells, and hemozoin in malaria-infected erythrocytes against a blood background and artifacts. Data from a *Plasmodium yoelii* murine model and cultured human *P. falciparum* were verified in vitro with confocal photothermal and fluorescent microscopy. With these techniques, we detected infected cells within 4 h after invasion, which makes hemozoin promising as a spectrally selective marker at the earliest stages of malaria progression. Along with the findings from our previous application of Cytophone with conventional lasers for the diagnosis of melanoma, bacteremia, sickle anemia, thrombosis, stroke, and abnormal hemoglobin forms, this current finding suggests the potential for the development of a portable rainbow Cytophone with multispectral laser diodes for the identification of these and other diseases.

## Introduction

Significant progress has been achieved in disease diagnosis using advanced lasers^1^. Among different laser methods, photoacoustic (PA) techniques have shown advantages in sensitivity, noninvasiveness, and depth penetration in biotissue up to 3 to 5 cm^2–4^. Several PA imaging devices have demonstrated promising clinical results in vivo in humans, including in the assessment of Crohn’s disease status^5^, diagnosis of breast cancer^6,7^, and monitoring of blood oxygenation in jugular veins^8^. The diagnosis of these and many other medical conditions often begin with the examination of a patient’s blood; however, existing blood tests are unable to provide early diagnosis because their sensitivity is limited by the small blood volume collected (typically 5–10 mL for venous and a few μL for capillary samples), which misses up to 10^3^ to 10^4^ of the abnormal cells or biomarkers in the entire blood volume (~ 5 L in adults)^9^. This large portion of missed cells can result in disease progression that is too difficult to treat, such as metastasis, sepsis, or stroke formed by circulating tumor cells (CTCs), bacteria, and clots, respectively^9,10^. In particular, despite enormous efforts in the development of diverse CTC assays, they are not yet recommended for clinical use because of their low sensitivity, inconsistent data, and as a result, have unclear diagnostic value, especially for early disease diagnosis^10,11^. In addition, most existing PA techniques, especially PA imaging, are unable to detect fast-moving, circulating disease biomarkers with typical velocities of 5 to 10 cm/s, even if present in subcutaneous vessels, further limiting their utility^10^.

These restrictions can be resolved by assessing larger blood volumes in vivo*,* using the principle of in vivo flow cytometry with fluorescent, photothermal (PT), Raman, and especially PA detection methods and their combinations^11–13^. Among these methods, in vivo PA flow cytometry (PAFC) has demonstrated clinical relevance for the direct detection in the bloodstream of bacteria (e.g., *S. aureus* and *E. coli*), clots, sickle cells, exosomes, different forms of hemoglobin (Hb) (e.g., oxy-, deoxy-, and metha-Hb), nanoparticles (NPs), and CTCs using intrinsic (e.g., melanin, Hb, Hz [hemozoin], cytochromes, carotenoids) or artificial (e.g., gold NPs) PA contrast agents^9,12,14–20^. In a clinical trial, PAFC offered an unprecedented ~ 1000-fold sensitivity improvement compared to existing diagnostic methods for detecting CTCs, clots, and CTC-clot emboli in melanoma patients in vivo^21^. We also demonstrated that in vivo PAFC has the potential for detecting circulating murine malaria-infected cells in an animal model at a 0.0000001% level of parasitemia^14,15^.

Nevertheless, the use of PAFC in resource-constrained countries is limited by the technical complexity, large size, and high cost of commonly used solid-state pulse lasers (e.g., flash-lamp, diode-pumped, Q-switched, and fiber-based)^1,12^. These limitations also make it extremely difficult to use an array of lasers with different wavelengths, which would aid in the spectral identification of biomarkers with different absorption spectra, also called spectral fingerprints, in particular, for NPs, Hb, and Hz^22–33^. Specifically, after our pioneering first application of spectrally tunable laser diodes in PA spectroscopy^34^, a few attempts were undertaken to use laser diodes for PA imaging^35,36^; however, the pulse energy of laser diodes was too low (at the level of a few microjoules [µJ]) to provide sufficient sensitivity. The pulse duration was also relatively long (90–120 ns) compared to the acoustic transit time across the small target to match the acoustic confinement for high-efficiency generation of PA signals^14,15^. Recently, significant progress has been made in the development of more powerful laser diodes with a pulse width of 15 to 30 ns and pulse energy of 0.1 to 2 mJ using emitter array sizes from 0.1 × 5 mm^2^ to 40 × 50 mm^37^. However, little progress has been made in the use of these lasers with PAFC. The goal of the work described here is to fill in the gap in this field of research by using an innovative PAFC-based rainbow (i.e., multicolor) Cytophone platform that integrates a miniature laser diode array with time-spectral light modulation, time-resolved detection of time-color-coded PA signals from each fast-moving cell(s) or biomarker(s), and high-speed signal processing. Compared with conventional laser sources, the advantages of the new laser diodes include their small size, use of compact emitter arrays with micro-optics, low cost, high repetition rate, necessary laser energy, and appropriate pulse duration.

To illustrate the unique performance of our rainbow Cytophone with laser diodes, we demonstrate that our novel platform has the ability to identify the unique spectra of multiple circulating objects, including white and red clots, mimic melanoma cells, and Hz alone and in malaria-infected red blood cells (RBCs) among blood background and other artifacts. Building on our previous malaria-related studies with solid-state lasers^14,15^, we now explore the potential of laser diodes as new promising optical sources to allow for increased PAFC specificity. This work required us to overcome challenges such as low pulse energy, high radiation divergence (especially with laser diode arrays), and high-speed detection and filtration of multicolor PA signals generated in fast-moving infected single cells by laser diode pulses with different wavelengths. We particularly focused our novel platform on malaria, which remains among the most significant infectious diseases, with nearly half of the world’s population at risk, resulting in 241 million clinical cases and 627,000 deaths in 2020 alone^22^. Over 96% of deaths occur in Africa, nearly all due to the *Plasmodium falciparum* species, though at least four other species account for human infections worldwide. Malaria represents a potentially ideal use case for PAFC, as invasion and growth of the parasite in RBCs is followed by the generation of iron-rich Hz, which can be detected by exploiting PA contrasts analogous to what is detected in melanoma CTCs^21^ given the similarities in optical and geometric properties between melanin and Hz^14^. The gold standard for malaria diagnosis has been light microscopy of blood samples, which allows for speciation and parasite quantification, though newer qualitative rapid diagnostic tests (RDTs) have quickly overtaken microscopy in endemic settings^23–33^. Unfortunately, both point-of-care diagnostics have limited sensitivity, with detection limits of 50 to 200 malaria-infected RBCs (iRBCs)/µL, take at least 20 to 30 min to perform, and RDTs are increasingly hampered by target antigen deletions. While conventional polymerase chain reaction (PCR) and especially RNA-based methods has improved sensitivity down to 1 parasite/µL or better, their utility in point-of-care settings is limited due to time, machinery, and consumables demands, as well as being prone to errors, as trace amounts of contaminant can lead to a misdiagnosis^23^.

The World Health Organization (WHO) seeks to reduce malaria-related deaths by at least 90% globally on or before 2030^22^, but currently, no malaria tests on the market have a reasonable cost or are sufficiently sensitive for identifying asymptomatic individuals, a reservoir that represents a critical obstacle in malaria eradication. Our work can address the WHO request by developing the required portable, noninvasive (needleless), label-free (no contrast agents), and safe (no skin damage, pain, or blood contamination concerns) device. Here we report the use of a novel rainbow (multicolor) Cytophone platform with small, cost-effective laser diodes for quick (in seconds) detection and real-time identification of *Plasmodium*-infected RBCs in vitro and in a murine model in vivo with a high potential for translation to humans.

## Results

### Design of the rainbow Cytophone platform

**Figure 1:**
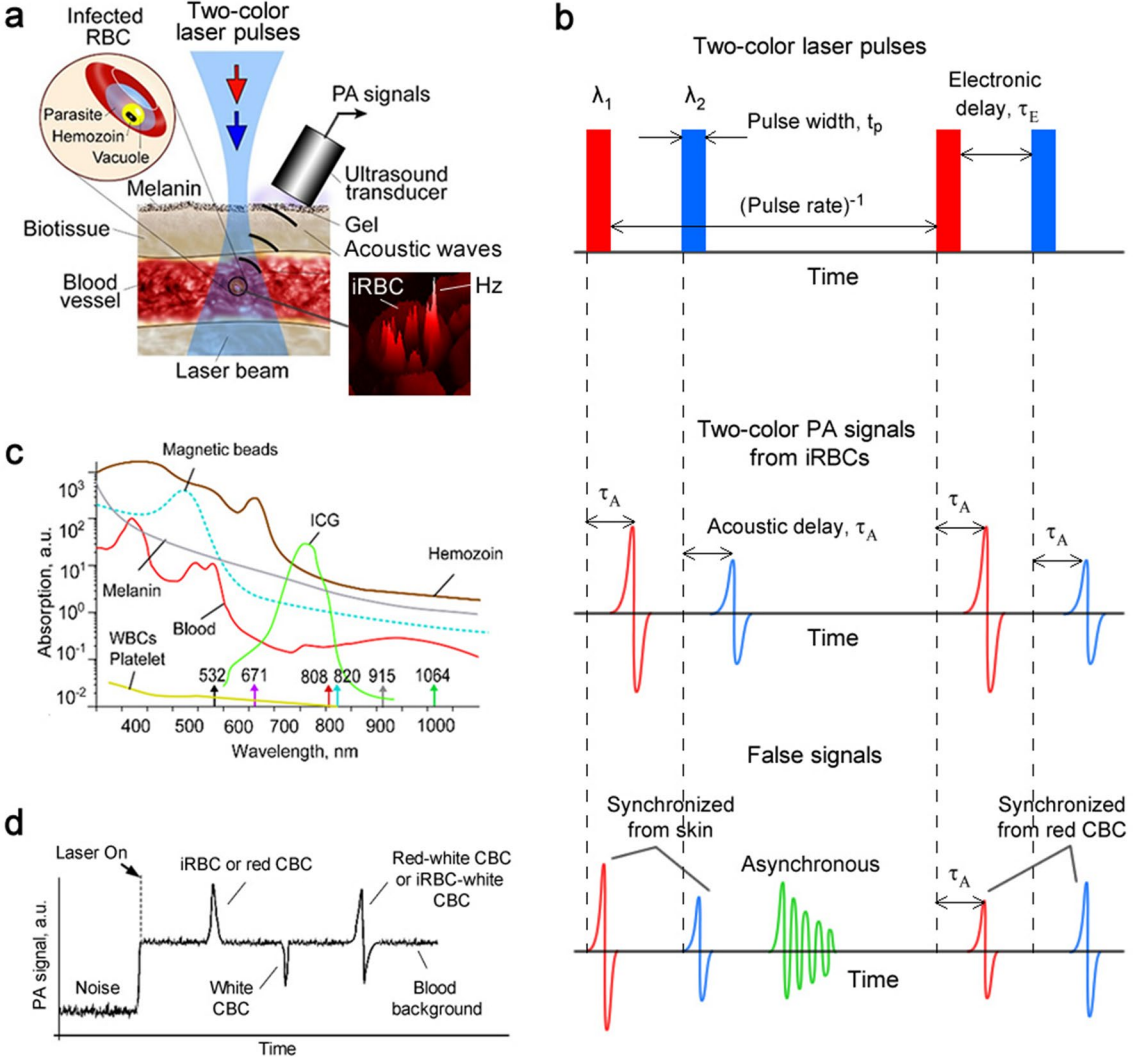
In vivo rainbow Cytophone diagnostic platform. (**a**) The principle of two-color Cytophone; insets show a schematic of iRBC structure (top, left) and a photothermal (PT) image of an actual iRBC (bottom, right) with Hz peaks. (Adobe Fireworks CS5, www.adobe.com) (**b**). Time-color coding of two high-repetition-rate laser pulses with different wavelengths of *λ*_1_ and *λ*_2_—(top)—for spectral identification of iRBCs (middle) in the background of synchronized (e.g., from pigmented skin) and unsynchronized false signals (bottom). (**c**) Absorption spectra of melanin, nRBCs, Hz, indocyanine green (ICG), and magnetic beads^14–21^. (**d**) PA traces with positive and negative PA contrast against a blood background created by many nRBCs and circulating blood clots (CBCs).

The distinctive feature of the novel rainbow Cytophone platform (Fig. 1a) is its use of a multispectral laser diode array that produces laser pulses with different wavelengths and the time delay for time-color coding (Fig. 1b). To validate this platform, we applied nanosecond (15–25 ns), high pulse repetition rate (1–3 kHz) laser diode pulses at two wavelengths, 808 nm and 915 nm. Transcutaneous laser irradiation of the vessels through intact skin leads to a temperature increase in the light-absorbing PA contrast agents, such as Hb in normal RBCs (nRBCs) and Hz with higher local absorption in iRBCs than in Hb in nRBCs in the near-infrared (NIR) region (Fig. 1c). The rapid thermal expansion of heated zones leads to the thermoacoustic generation of acoustic waves referred to as PA signals that are detected with a small (a few mm in diameter) ultrasound (US) transducer (Fig. 1a).

PA signals from Hb in nRBCs in the detection volume create a trace baseline (Fig. 1d). When iRBCs with a higher Hz-related local optical absorption above the Hb background of nRBCs pass the detection (i.e., irradiated) volume, the corresponding absorption increases, generating sharp positive transient PA peaks over the blood background. For comparison, white and red circulating blood clots (CBCs) produce negative and positive PA peaks (Fig. 1d).

Based on the distinctive features of the absorption spectra of Hz in iRBCs and Hb in nRBCs, specifically their differential absorption in the 770 to 930 nm spectral window, we selected two-color mode to demonstrate the ability of Cytophone to spectrally identify iRBCs with two laser pulses at 808 and 915 nm and a short electronic time delay τ_E_ (10 μs) for time-color coding (Fig. 1b, top). These pulses with a repetition rate of 3 kHz interact with the same fast-moving individual cells, which generates time-color coded PA signals from the same cells when they pass the detection volume (Fig. 1a). Specifically, iRBCs produce PA signals with higher amplitudes at 808 nm than at 915 nm (Fig. 1b, middle), while nRBCs generate PA signals with higher amplitudes at 915 nm than at 808 nm like for red CBCs (Fig. 1b, bottom, right). The combination of time-color laser pulse coding with the time-resolved PA signal detection also allows the identification of a pair of synchronized true PA signals from iRBC in the vessel coming to the transducer (Fig. 1a) with a well-resolved acoustic time delay τ_A_ (≥ 0.2 µs) compared to paired synchronized background signals from the pigmented skin layer (Fig. 1b, bottom, left) as well as non-synchronized with laser pulses the false signals (Fig. 1b, bottom, center) of different origins (e.g., from vibration, environmental acoustics, and electromagnetic interference).

**Figure 2:**
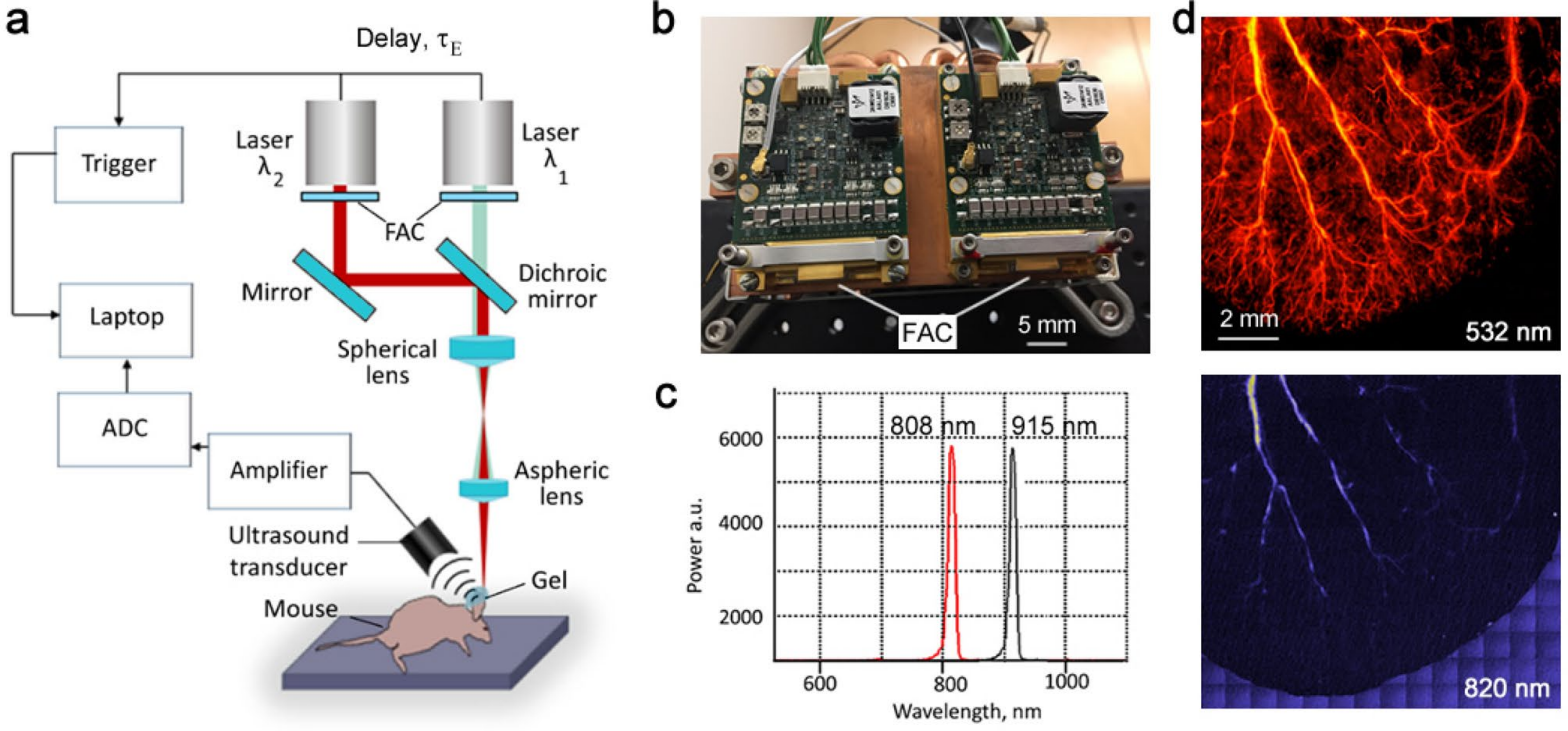
In vivo two-color Cytophone setup with two laser diodes. (**a**) Optical scheme. (Adobe Fireworks CS5, www.adobe.com) (**b**) Photo of two laser diode modules with two wavelengths. (**c**) Spectra of laser diode emission with central wavelengths of 808 and 915 nm. (**d**) PA microscope images of mouse ear fragment at 532 nm (top) and 820 nm (bottom). FAC = fast-axis collimation system; ADC = analog-to-digital converter.

In the Cytophone, after a so-called fast-axis collimation (FAC) system (Fig. 2a,b) using a microlens array, the beams from two laser diodes are mixed in the coaxial direction with a dichroic mirror (Fig. 2a). The focusing optics, consisting of a spherical and cylindrical lens, forms a linear beam with a width of approximately 80 µm and a length of 1 mm. The two compact emitter arrays (Fig. 2b) provide two wavelength pulses (Fig. 2c) with an energy of 110 to 130 µJ. Light scattering in biotissue results in laser beam blurring that significantly decreases the optical resolution (Fig. 1a). To provide higher spatial resolution, we applied the acoustic resolution Cytophone schematic using the focused cylindrical US transducer with a lateral resolution of 65 ± 9 µm. The cylindrical transducer configuration allows us to minimize the detection volume and, thus, blood background from nRBCs, thereby maximizing the signal-to-noise ratio (SNR) from iRBCs while simultaneously assessing all iRBCs in the whole blood vessel cross-section. To collect PA signals with the typical bipolar waveforms (Fig. 1b, middle), the platform was equipped with an analog-to-digital converter board. PA signal waveforms were averaged from 4 to 16 times to increase SNR, then transformed into traces in analogous to conventional flow cytometry^12^ (Fig. 1d). As a result, Cytophone generates trace peaks with positive and negative contrasts against a blood background (Fig. 1d). The characteristics (e.g., amplitude and width) for each peak above the established background level were then analyzed by using customized MATLAB-based software.

To explore whether the acoustic resolution Cytophone platform with advanced laser diodes can provide both diagnostic value and high spectral specificity, we compared its performance in vivo and in vitro to conventional PAFC, which utilizes solid lasers at wavelengths of 532, 671, 820, and 1,064 nm*.* To do so, we used our advanced vessel phantoms^38,39^ and disease-related PAFC contrast agents, including magnetic beads mimicking melanoma CTCs^38^. The agents were intravenously or peritoneally delivered in the mouse body, followed by PA detection in 40 to 60 µm diameter ear vessels (Fig. 2d). The PA contrast of blood vessels at 820 nm was lower than at 532 nm (~ 20-fold), which correlates with the absorption spectrum of Hb (Fig. 1c). This spectral signature increases the PA contrast of iRBCs above blood background because Hz absorption in iRBCs only slightly decreased.

### In vitro validation of the rainbow Cytophone platform

The two-color Cytophone with two wavelength laser diodes was initially validated in vitro through spectral identification of traditional PAFC contrast agents in dynamic conditions using our dynamic blood vessel phantom with fast-moving blood cells, CTCs, and clot phantoms under well-controllable flow conditions^38,39^. This phantom was composed of polyvinyl chloride plastisol with TiO_2_ nanoparticles modeling biotissue with medically-adequate scattering–absorbing properties, a melanin surface layer mimicking the pigmented skin, plastic tubes with different diameters and depths mimicking blood vessels, and a pump to move cell phantoms and real cells with velocities of 0.5–5 cm/s. To mimic flowing cells and artifacts which can produce false signals, we tested (1) 8-µm magnetic beads with absorption in the NIR range, similar to that of melanoma cells (Fig. 1c); (2) RBC aggregates in human blood mimicking red CBCs; 3) 100-µm optically transparent silica beads as white CBCs phantoms; (4) indocyanine green (ICG) dye to provide a bulk absorption background with ICG aggregates as red CBC phantoms; and (5) commercially available Hz.

**Figure 3:**
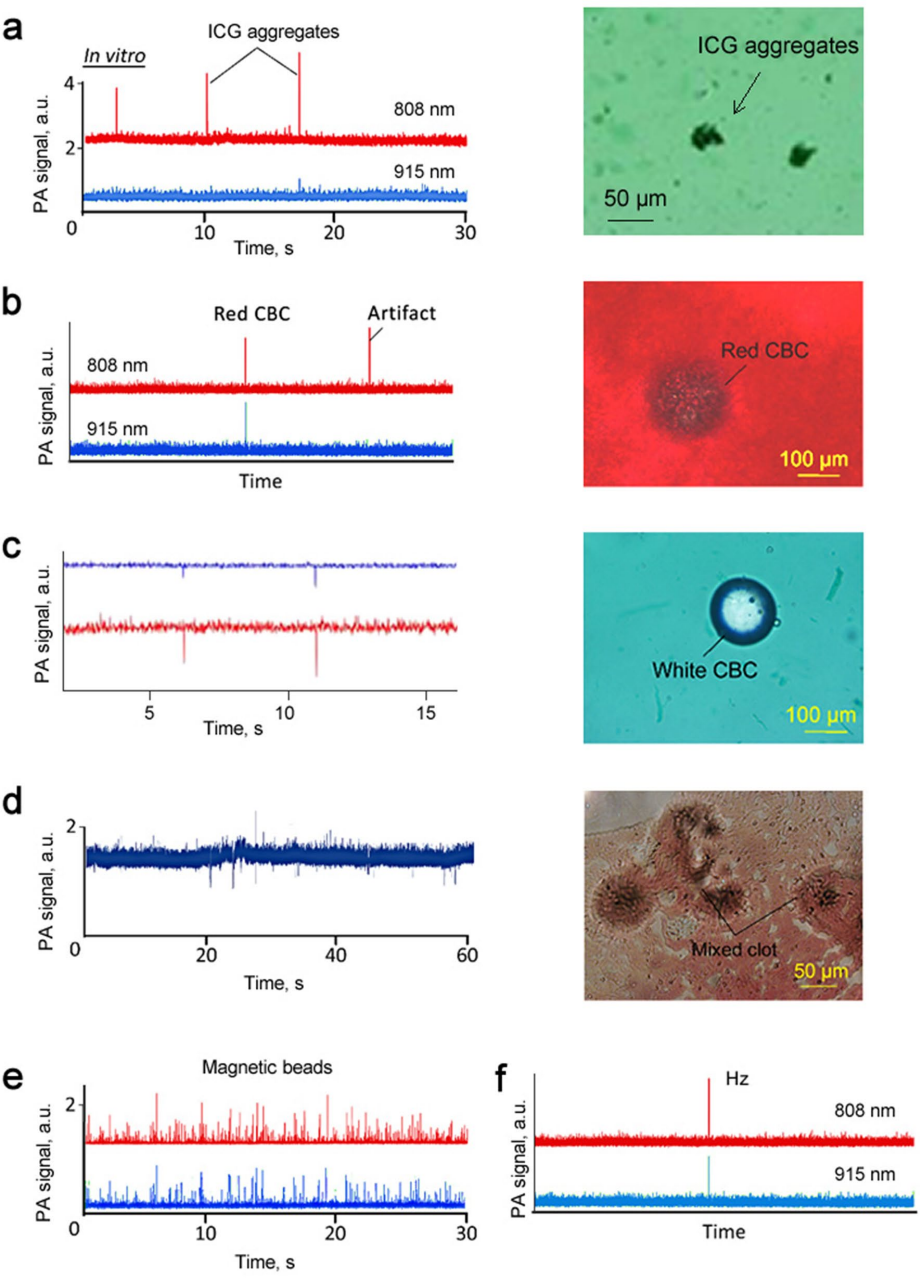
Validation of two-color (808 nm/915 nm) Cytophone performance through spectral identification of conventional PA targets with positive and negative contrasts in vitro using the dynamic vessel phantom. (**a**) PA traces with the positive contrast peaks from rare indocyanine green (ICG) aggregates in relatively homogenous ICG solution (left) and bright-field image of ICG aggregate (right). (**b**) Two color PA traces from RBC aggregates in blood with “one color” artifact (left) and bright-field image of RBC aggregate (right). (**c**) Negative contrast PA peaks from silica beads as white circulating blood clot (CBC) phantoms (left) and bright-field image of a single bead (right). (**d**) Positive and negative contrast PA peaks from red and white CBCs in blood (left) and bright-field image of mixed red-white CBCs in the blood (right). (**e**) Two-color positive contrast PA signals from 23-µm magnetic beads in the background of many 8-µm magnetic beads. (**f**) Two-color positive PA peaks from synthetic ~ 200 µm Hz.

In our initial study, we used ICG dye with a well-characterized absorption spectrum at a maximum wavelength near 808 nm (Fig. 1c) to estimate two-color Cytophone performance. Using conventional bright-field microscopy, we identified the presence of small ICG aggregates in a relatively homogenous solution (Fig. 3a, right). The higher local absorption of ICG aggregates above the ICG absorption background solution led to the generation of sharp positive PA peaks above the baseline in flow with a velocity of 1 cm/s in a 1-mm diameter vessel phantom (Fig. 3a, left). These peaks appeared preferentially at 808 nm as the optical absorption of ICG at 915 nm (second color used) is approximately 9 times lower than at 808 nm (Fig. 1c).

Next, we obtained two-color PA peaks (Fig. 3b, left) from RBC aggregates in blood (Fig. 3b, right). The PA peak amplitudes at 808 nm and 915 nm are consistent with the RBC absorption spectrum (Fig. 1c). We also observed a single peak at 808 nm, which is associated with added ICG aggregate. These data suggest that a two-color Cytophone has the potential to identify a selected target (i.e., red CBCs) in the presence of other artifacts based on specific fingerprints.

To further assess the two-color Cytophone’s performance, we estimated its capability to identify white CBCs that provide negative contrast peaks due to lower absorbance than nRBC-related backgrounds (Fig. 1d). A suspension was created by adding 30 mg of 100-µm diameter silica beads (Fig. 3c, right) to 1.5 mL of phosphate-buffered saline (PBS), and the suspension was shaken every 15 min to prevent microparticle sedimentation and aggregation. We then added ICG solution to model a blood background. We observed two-color (808 nm/915 nm), narrow, negative-contrast PA peaks associated with the sudden drop in absorbance in the detection volume as individual transparent silica beads passed through the laser beam area (Fig. 3c, left). Mixed red and white CBCs in human blood (Fig. 3d, right) created positive and negative contrast PA peaks at 915 nm (Fig. 3d, left). In the past, we have utilized nanosecond solid lasers to obtain comparable negative PA contrasts^16,46^ using similar CBC parameters. The similarity in these results indicates the possibility of replacing solid lasers with laser diodes for the same application.

Next, we used relatively rare 23-µm magnetic beads in the background of many 8-µm magnetic beads (40 µL of 1% beads in 3,960 µL of PBS), to serve as surrogates of CTC and nRBC phantoms^38^, respectively. We observed many two-color (808 nm/915 nm) transient narrow positive peaks from the individual relatively large magnetic beads above the “blood background” modeled by small beads (Fig. 3e). The PA peak amplitudes (red, 808 nm; blue, 915 nm) match the absorption spectrum of the magnetic microparticles (Fig. 1c). These data are very similar in SNRs in the range of 5 to 12 measured previously with a 1,064 nm laser under similar conditions^38^. Again, this comparable sensitivity indicates the possibility of using the laser diodes in a similar application.

Finally, to verify the potential of laser diodes to detect malaria-associated Hz, we attempted in vitro detection of commercially available Hz, whose optical absorption spectra and morphology (size and shape) (Fig. S1) are similar to native Hz^30–33^. Our results demonstrate that Cytophone can better detect Hz with PA signal amplitudes at 808 nm than at 915 nm (Fig. 3f), which correlates with the Hz absorption spectrum (Fig. 1c).

In conclusion, using in vitro flow conditions, we demonstrate that the Cytophone platform with laser diodes has the potential to detect and identify multiples targets (e.g., ICG aggregates, magnetic beads, white and red CBCs, and Hz).

### In vivo detection of *Plasmodium*-iRBCs with two-color Cytophone

**Figure 4:**
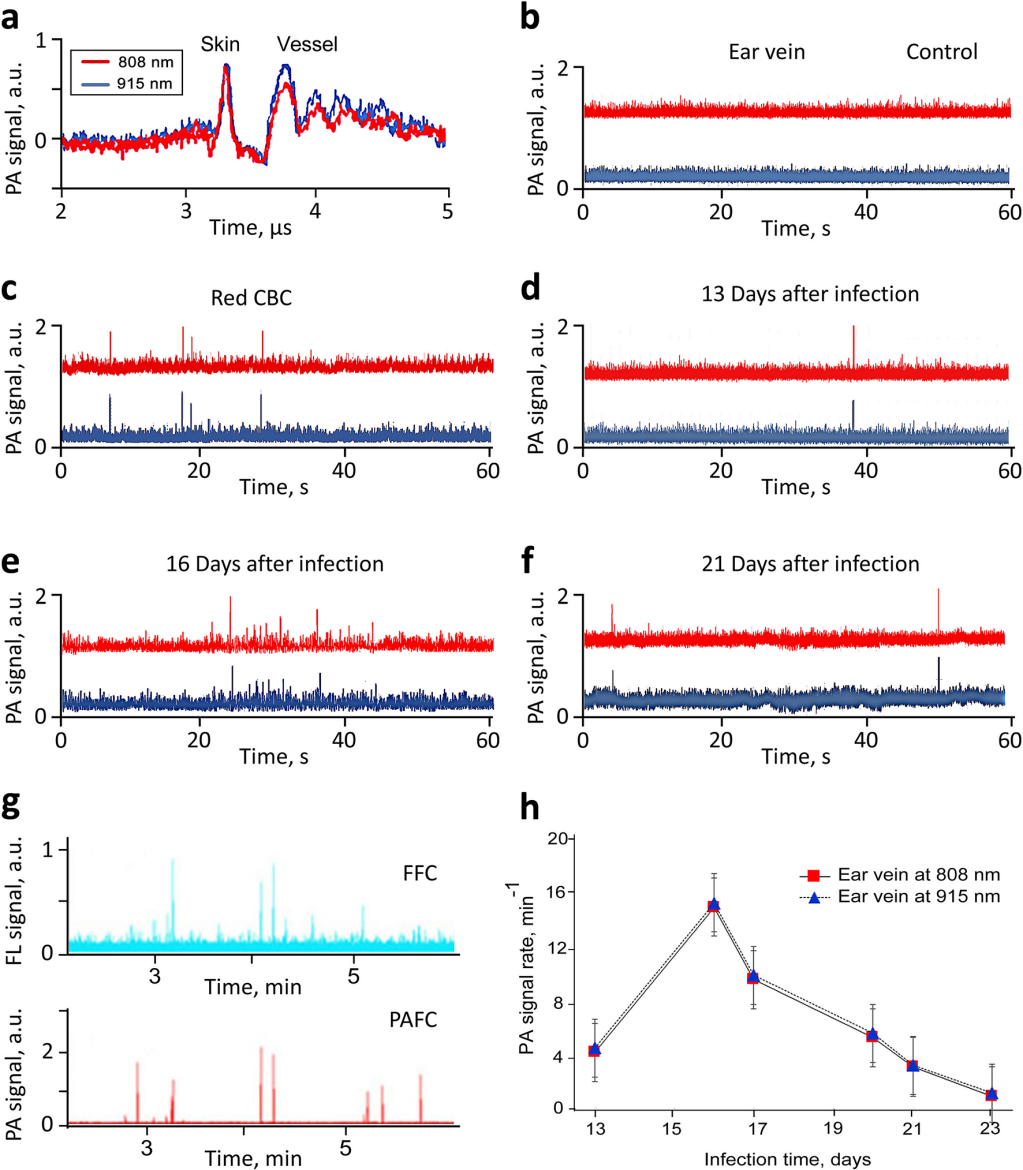
In vivo monitoring of two-color (808 nm / 915 nm) PA traces in control and malaria-infected mice with *P. yoelii* GFP^+^ parasites. (**a**) Time-resolved detection of PA signal waveform in a control mouse from an ear vessel as compared to skin. (**b**) In vivo PA traces from a healthy (control) mouse. (**c**) In vivo PA traces from an uninfected mouse with RBC aggregates. In vivo PA traces from an infected mouse at 13 (**d**), 16 (**e**), and 21 (**f**) days after infection. (**g**) In vivo verification of laser diode-based Cytophone data by testing the same infected mouse using PAFFC platform integrating PAFC (bottom trace) and fluorescent flow cytometry (FFC) module (top trace). (**h**) In vivo dependence of the PA rates (number of PA signals per minute) on time after infection.

After comprehensive verification in vitro with contrast agents that are well-established for PA techniques, we tested Cytophone performance in vivo by monitoring PA signals from ear microvessels in control and infected mice (Fig. 2d). Data collected from the first cohort of 5 healthy mice were used to further optimize parameters. The PA probe, which consists of an optical focusing tip and US-focused transducer, was positioned on the ear’s skin above the selected vein and standard US gel provided acoustic coupling. The navigation of the transducer focal volume on the examined vein was accurately controlled by optical imaging and monitoring of PA signals as the PA probe scanned in different directions (Fig. S2). The PA signal amplitudes from the selected veins were 1.65 ± 0.32 (SD) times higher than the background signals from the skin. PA signals with typical widths of 0.06–0.1 µs (Fig. 4a) came from the vessels with a typical depth of 200 to 250 µm to the transducer with a well-distinguished acoustic time delay (0.15–0.3 µs). This allowed us to easily eliminate the influence of PA background signals from the skin on the Cytophone’s sensitivity by time-resolved PA detection of signals from circulating objects only. As a result, no transient positive PA signals above the established blood background at two wavelengths were observed in control mice (Fig. 4b). The low amplitude fluctuation of the blood background (baseline), typically 3% to 5%, depends on the fluctuation of the laser energy, electrical and acoustic noise, vibration, physiological motion, and slight fluctuation in the number of nRBCs in the detection volume. The number of nRBCs is more profound in small vessels (10–15 µm).

We next tested the Cytophone’s ability to detect RBC aggregates (i.e., red Hb-rich CBCs) in vivo. Using well-established procedures^12,21,46^ and low temperature, we created RBC aggregates that were identified with high-speed optical imaging^46^. The aggregates moving through the detection (irradiated) volume created transient positive PA peaks simultaneously in two-color channels (Fig. 4c). The amplitudes at the same laser energy were a little higher (15–20%) at 915 nm than at 808 nm in line with blood absorption spectra (Fig. 1c).

Next, GFP-expressing *P. yoelii* were intraperitoneally injected into the mice^14^. Parasitemia was evaluated by monitoring the PA signal amplitudes and rates (number of PA signals per time unit) daily over approximately one month (see selected examples in Fig. 4d–f). To verify data obtained with laser diodes, we tested the same mice with our more established integrated PAFC and fluorescence flow cytometry (FFC) system (referred to as PAFFC)^14^ using a pulsed solid laser at a wavelength of 671 nm to generate PA signals as well as a continuous wave (CW) laser at 488 nm to excite emission in GFP-expressing parasites. Multimodal PAFFC capacity allowed us to identify iRBCs through the temporal coincidence of FL and PA signals from parasites containing both GFP and Hz inside iRBCs, respectively (Fig. 4g). The presence of a PA peak only (i.e., not FL-related peaks) indicated that the Hz was alone in blood plasma or engulfed Hz in WBCs (e.g., neutrophils)^14,26^.

In general, PAFFC confirmed the Cytophone data indicating the presence of iRBCs in the same infected mice. Monitoring PA signals for one month in infected mice revealed the following tendency during malaria progression: (1) gradual signal rate increase during the first 15 (we started on Day 13) to 16 days; 2) reaching a maximum at 15 to 17 days; and (3) eventual decline to full disappearance by 28–30 days (Fig. 4h) that is in line with our previous study^14,15^. This pattern is well described and is related to immune-mediated clearance in the mouse model^14^. The data obtained with laser diodes are in line with the data obtained with solid lasers^14,18^, with some slightly different features depending on the vessel location, flow rate, and immune response.

In vivo PA monitoring of artificial Hz, magnetic NPs, and B16F10 melanoma cells injected into a tail vein revealed that magnetic NPs and melanoma cells (Fig. S3) were cleared relatively quickly (20 min to a few hours), which is consistent with our previous results^9,12^. During the PA probe scanning along the vessels, we observed stationary PA signals with a random spatial distribution. These signals were relatively stable in amplitude and were well-distinguishable from sharp (i.e., short-lasting) transient PA peaks produced by fast-moving iRBCs. This finding suggests the possible presence of sequestered iRBCs (i.e., adhered to vessel walls). Of note, sequestration of iRBCs to endothelial vessels and other tissues is a well-described phenomenon in malaria^23,25^.

### Ex vivo analysis of *P. yoelii*-iRBCs with Cytophone

**Figure 5:**
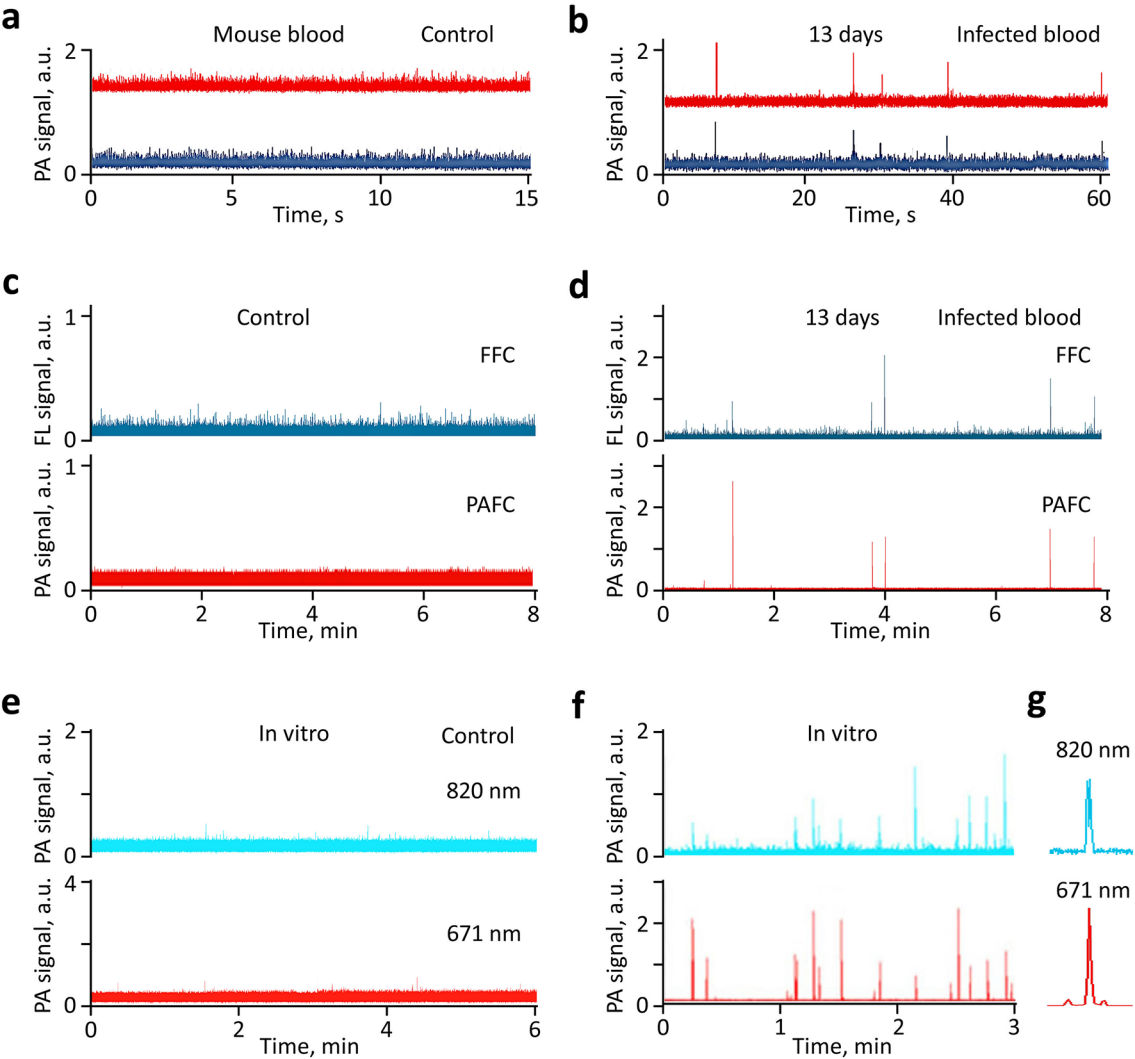
Ex vivo verification of in vivo Cytotophone data using the blood vessel phantom. Two-color PA trace from (**a**) control (uninfected mouse) and (**b**) *GFP* + *P. yoelii*-infected mouse blood at 13 days post-infection using Cytophone with two laser diodes at 808 nm and 915 nm. (**c**) Fluorescent and PA traces from control blood using PAFFC platform (integrating PAFC and FFC modules) with pulse lasers at 671 nm and continuous-wave lasers at 488 nm for generation of PA and fluorescent (FL) signals. (**d**) Ex vivo fluorescent and PA trace from GFP^+^ *P. yoelii*-infected mouse blood at day 13 using PAFFC platform. Two-color PA traces were obtained with a two-color Cytophone platform using lasers at 671 nm and 820 nm from (**e**) control human blood and (**f**) *P. falciparum-*iRBCs from in vitro culture. (**g**) Fragment of trace, demonstrating the temporal coincidence of PA peaks at different wavelengths from the same iRBC.

Next, we analyzed ex vivo samples from *Plasmodium*-infected mice with the same Cytophone/laser diodes and PAFFC/solid laser platforms using our dynamic blood vessel phantom with flowing blood samples^38,39^. No signals above established noise levels were observed in blood samples from the control blood samples (i.e., uninfected mice) either with the two-color Cytophone (Fig. 5a) or multimodal PAFFC (Fig. 5c). In contrast, both Hz-related PA signals and GFP-associated FL signals from iRBCs with GFP-expressing *P. yoelii* were observed with a similar rate at the same infection stage as seen with paired in vivo data. Specifically, the temporal coincidence of two-color PA peaks (Fig. 5b) correlated with the absorption spectra of Hz (Fig. 1c), clearly indicating the Hz-related origin of the PA signals. Moreover, the temporal coincidence of PA and FL signals (Fig. 5d) confirms this conclusion, considering that Hz should be located within GFP-expressing *P. yoelii-*iRBCs. Hz-associated PA signal amplitudes sufficient for identification were induced by laser diodes above background noise (Fig. 5b) and were comparable with those induced by solid lasers (Fig. 5d). This validates Cytophone’s ability to detect *Plasmodium*-iRBCs. In addition, the SNR for PA channels was typically higher than in FL channels, which indicates the advantage of Cytophone as a diagnostic platform compared to FFC modules. Additionally, FFC requires labeling of iRBCs in vivo*,* which is not easy, and most FL labels are toxic for humans.

### In vitro PA and PT characterization of hemozoin

**Figure 6:**
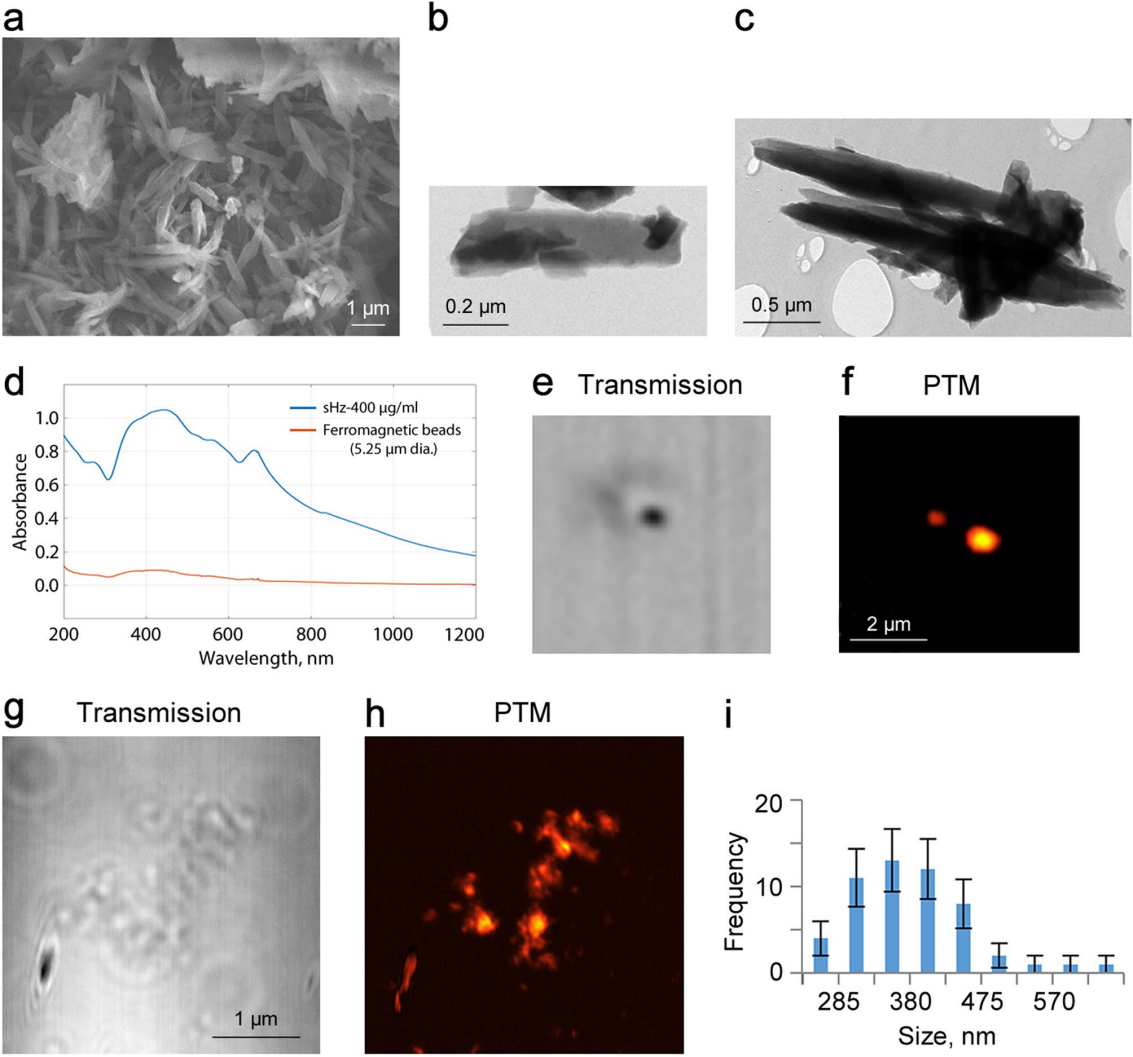
Characterization of hemozoin (Hz) alone used for calibration of Cytophone and PTM systems. (**a**) STEM image of Hz clusters. TEM images of a single (**b**) and clustered (**c**) Hz. (**d**) Absorption spectrum of synthetic Hz. Spectral absorbance of Hz and for comparison magnetic microparticles with a diameter of 5.5 µm. Transmission/bright field (**e**) & (**g**) and photothermal (**f)** & (**h**) images of small individual Hz at low concertation (5 µg/mL) after ultrasound treatment (24 kHz, 5 min) and clustered Hz at high concertation (25 µg/mL). (**i**) Histogram of Hz crystal size distribution obtained with photothermal microscopy (PTM); error bars present standard deviation, *n* = 55.

To verify the PA data, we also applied confocal photothermal microscopy (PTM)^40–45^, a technique we have developed, which has very similar physical principles to PA techniques (i.e., transformation of absorbed light energy into heat) but with improved in vitro imaging resolution^40,41,45^. While Cytophone detects PA waves because of thermal expansion of heated zones, in PTM, the thermal effects are detected through defocusing of the probe beam due to the changes in the temperature sensitive refractive index^40–45^. The advantages of PTM in vitro, as compared to other optical techniques, are its improved sensitivity and spatial resolution^41^, allowing us to verify the Cytophone’s capability to identify single Hz nanocrystals by their PTM-based visualization alone or within cells. As mentioned, we used commercially available Hz with properties similar to natural Hz^30–33^. TEM, STEM, and EDS instruments (Fig. 6a–c; Supplementary Fig. 1Sa–d) demonstrated high heterogeneity in Hz size and shape, starting during the initial nucleation process as non-ideal cube-spherical nanocrystals with a minimal size of 60 to 80 nm and transforming into elongated structures with a size ratio (long to short side) of 2:1, and mostly 3:1 with maximal length up to 1 mm. During their growth, single nanocrystals can form a chain of individual nanocrystals (“linear cluster”). At high concentration, Hz is frequently aggregated, with an average Hz cluster size between 250 to 500 nm with a median of 320 nm. Unlike TEM and STEM, PTM does not typically require highly specific, time-consuming preparation (just a few minutes). With a 40 × objective lens, PTM images of Hz showed similar results, including some heterogeneity (Figs. 6e-g) with a quite symmetrical size distribution around a median value of 330 nm (Fig. 6i). After ultrasound disintegration of Hz clusters, PTM with a 100 × objective lens was able to distinguish individual Hz nanocrystals with diffraction-limited resolution at a level of 220 nm (Fig. 6f). These nanocrystals provided well-detectable PA signals (e.g., Figs. 3f and S3a) with amplitudes comparable to or higher than melanin nanoparticles in melanoma cells (Fig. S3b, c). Because using PA and PT techniques to study melanoma is well-established^21^, these techniques can be easily adopted for malaria detection with laser diodes that can replace traditional laser sources.

### Ex vivo imaging of *P. yoelii*-iRBCs at different infection stages

**Figure 7:**
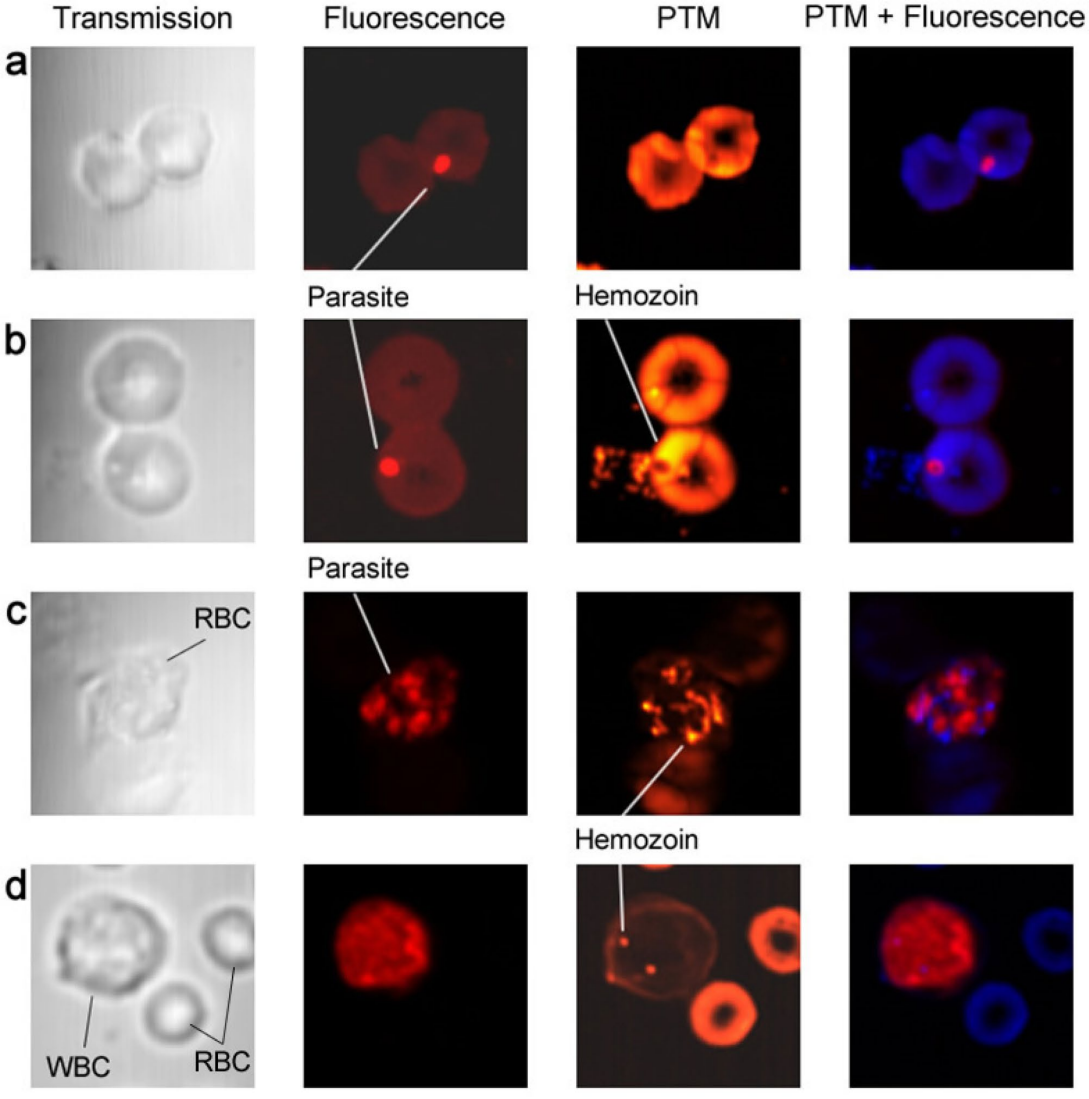
The optical images of control and infected with *P. yoelii* cells stained with propidium iodide (PI). Column: Transmission (first), fluorescent (second), photothermal microscopy (PTM) (third), and integrated (fourth) images of mouse blood cells. (**a**) Early ring stage (2–4 h after infection) with a single parasite in RBC with no sign of Hz yet. (**b**) Trophozoite stage (6–10 h after infection) with a single parasite in RBC with one or few Hz. (**c**) Maturated schizont stage (24 h after infection) with many parasites and Hz. (**d**) Hz in WBC with no signs of parasites and Hz in RBCs (24 h after infection).

After PA and PT characterization of synthetic Hz, we collected blood samples from C57BL/6 mice infected intraperitoneally with a GFP-expressing *P. yoelii* 17XNL*.* Transmission (bright field), fluorescent, and PTM techniques were applied to image the individual cells in blood samples stained with propidium iodide (PI) for selective targeting of parasites at different stages of development from early rings to mature schizonts (Fig. 7). While transmission images provided little to no structural information on the parasites and Hz inside iRBCs, fluorescent (FL) and PTM confocal platforms in combination clearly distinguished PI-labeled parasites and Hz, respectively, among autofluorescent and Hb-associated absorption backgrounds within RBCs. Specifically, integrated confocal PTM and confocal FL microscopy showed both Hz and parasite co-localization in *P. yoelii*-infected mouse RBCs with good resolution and high imaging contrast. PTM allowed visualization of the intracellular distribution of Hz and their aggregates (Fig. 7b,c; Fig. S4c–e), which are not visible with conventional transmission microscopy (Fig. 7a). Within 2 to 4 h of invasion, RBC shape was transformed from the traditional biconcave discoid to a smaller spherical shape. At the trophozoite stage, the parasites almost completely consumed Hb content, leading to the formation of multiple Hz crystals, which were visible in RBCs with reduced or even no Hb background compared to nRBCs (Fig. 7c, Fig. S4c). Only PTM was able to monitor the intercellular details of parasite progression from the initial ring stages (Fig. 7a,b) to the schizont stage accompanied by the release of Hz from RBCs into plasma (Fig. S4f). In some blood samples, we were able to observe WBCs with ingested Hz (Fig. 7d) compared to control with no Hz (Fig. S4a).

### In vitro imaging of *P. falciparum*-iRBCs

Our studies of murine malaria, both in vivo and ex vivo, successfully demonstrated the capability of the novel rainbow platform to detect and resolve *Plasmodium*-derived Hz within RBCs, so we next tested the rainbow Cytophone on the human pathogen, *P. falciparum*. Frozen samples of the *P. falciparum* strain 3D7 were thawed using a multi-step established procedure (see Methods)^23–26^. The parasite cultures were synchronized with a 5% sorbitol solution to include only 0 to 12-h post-infection ring-stage parasites. Nine total samples were taken of the synchronized cultures, including the following time points after erythrocyte infection: 0–4 h, 6–10 h, 12–16 h, 18–22 h, 24–28 h, 30–34 h, 36–40 h, 42–46 h and 48–52 h.

No signals were observed in uninfected control blood samples (Fig. 5e). Using in vitro cultured parasites*, P. falciparum*-induced Hz in iRBCs (Fig. 5f,g) generated larger PA signals than ex vivo* P. yoelii*-infected RBCs (Fig. 5b,d), indicating the promising clinical potential of Cytophone for malaria diagnosis. Thus, data indicate that the replacement of solid lasers with the more cost-effective and smaller multicolor laser diodes are suitable for the detection of malaria parasites.

To investigate the appearance of *P. falciparum* parasites at different erythrocytic life cycle stages, we again employed transmission (bright field), fluorescent, and PTM techniques to individual cells in culture samples in blood stained with PI (Fig. S5). Images were obtained prior to RBC invasion (a), soon after invasion (within 4 h) with the formation of single and clustered Hz (b, c), matured stages (24 h after infection) with multiple parasites likely schizonts and Hz (d), and a unique case of a released parasite with Hz inside (e). It should be emphasized that we were able to identify Hz formation events at the earliest stages of invasion (within 4 h). PTM data can also be presented in 3-D simulation (Fig. S6) as well as video with 3-D rotation (Supplementary Video), which enables more accurate spatial localization of Hz with high-resolution PTM. PT data correlated well with conventional staining of *P. falciparum* slides at different time points from 0–4 h to 48–52 h (Fig. S7).

## Discussion

In this work, we demonstrate for the first time that our two-color Cytophone with advanced laser diodes has the potential for further development as a novel diagnostic platform for the detection and identification of *Plasmodium*-iRBCs, as well as several other intrinsic PA markers associated with other diseases of global importance. We demonstrated that a Cytophone with two small laser diodes (wavelengths: 808 nm and 915 nm) can identify Hz, a heme degradation by-product generated by all known *Plasmodium* species, against a blood background and artifacts. Uninfected blood provided PA traces with no transient sharp PA peaks above the blood background (Fig. 4b, 5a,c,e), while blood infected with different *Plasmodium* species, including in vitro cultured *P. falciparum*, the most prevalent and virulent human species, (Figs. 4d–f, 5b) clearly show the possibility for two-color (e.g., 808 nm/915 nm or 671 nm/820 nm) identification of Hz-associated peaks with a specific PA signal ratio against a background of normal blood or artifacts with other ratios and/or typically produced signals at only one wavelength. The absorption spectra of Hz demonstrated gradually decreased absorption with an increased wavelength in the NIR range with a slight maximum at 670 ± 2 nm, while RBC absorption is slightly increased in this range (e.g., 700–950 nm [Fig. 1c])^32^. Quantitative data of parasitemia can be obtained by counting PA signal numbers per time unit (i.e., signal rate) that are associated with individual iRBCs for a certain time divided by the blood volume passing the detection point for the same time. The flow rate to calculate a blood volume can be roughly estimated for an examined vessel from the literature^15^ or measured directly by US^15^ and/or PA techniques^9^.

We have additionally demonstrated that our integration of PTM and PAFC allowed for the detection of tiny (approximately 100 nm) single Hz nanocrystals formed only a few hours (4 h) after parasite (merozoite) invasion of RBCs, as well as Hz aggregates (Fig. 7, S4-6). Using conventional detection techniques, Hz was thought to be detectable only after 12 h or more for up to 24 h. Only magneto-optical (MO) methods have demonstrated a potential to detect Hz formation during 6 -10 hours^46^. Moreover, according to a recent analysis^47^, the rate of in vivo crystalization, an important step in Hz biosynthesis, is estimated as 7,000 hemes/s. Taking into account that there are approximately 6 to 10 million hemes in a single Hz, Hz formation could theoretically begin as soon as 15 to 24 min after invasion. If our data push back the timing of Hz formation in iRBCs, we may need to reconsider the physiology of early parasite growth immediately after invasion, as well as the potential utility of advanced PTM and Cytophone platforms to understand the dynamics of Hz over the full 48-h life cycle. We suggest that the Cytophone may have the potential to diagnose malaria early in the course of blood stage infection^14,15^, as well as at low parasite densities, while also potentially being useful in drug efficacy and viability studies *in vivo*^23^.

We found that the lifespan of Hz in circulation is relatively long (up to 24 h before its notable clearance by the reticuloendothelial system without signal degradation) (Fig. S2), while other agents (e.g., melanin NPs, melanoma cells, dyes, and bacteria) are typically cleared in a much shorter time (10 min to a few hours)^9^. Biologically generated Hz kinetics have been explored in murine and human malaria, and have demonstrated that Hz, once released, is rapidly phagocytized, with intracellular Hz present in these phagocytes until they leave the circulation. Notably, our integrated multimodal PAFFC platform allows for the identification of the parasite and Hz location in iRBCs through temporal coincidence of corresponding signals or extracellularly in plasma through the presence of single signals from Hz (Figs. 4g, 5d)^14^. We believe that the same platform can be used for the identification of Hz in monocytes and neutrophils after post-schizont rupture of iRBCs, subsequently leading to the release of free Hz in plasma and then its engulfment by phagocytes (Fig. 7d). In particular, neutrophils and monocytes can be identified through labeling with antibodies against CD11 and CD15 markers, respectively, or in combination. While these uses are intended for animal models, it is hopeful that in the future, conjugated ICG or low-toxicity gold nanoparticles approved for a pilot study in humans^20^ can be used for localization of Hz in different vascular compartments. In addition, we aim to leverage these data to optimize in vivo PAFC capacity to distinguish intraerythrocytic Hz from free Hz and phagocytized Hz. On the other hand, the clearance of free Hz from circulation takes a few days only^48^, which minimizes the risk of false positivity.

As we noted, using a spatially scanning laser beam, the Cytophone platform may identify the spatial distribution of iRBCs adhered to the vessel wall. While our data are preliminary, we hypothesize that this may represent a well-known phenomenon central to malaria pathogenesis, particularly in *P. falciparum*, known as sequestration. Sequestration of iRBCs occurs in the peripheral vasculature, as well as in several tissues, and while this has been extensively described in murine and human post-mortem studies, limited data have demonstrated such processes occurring in vivo without the use of labeling. Further work is ongoing in this area, including comparing results of iRBC counts in veins and arteries^15^.

Perhaps most critical in the further development of the novel Cytophone platform would be the ability of the device to differentiate between *Plasmodium* species. Because different species can have slightly different absorption spectra, it might be possible to differentiate between *Plasmodium* species via selection of the proper laser wavelength. In addition, different species produce different Hz nanocrystal sizes and shapes^33^. Because PA signal waveform shape depends on target size^2,3^, this may be another way to identify different species. While we have not yet tested the new Cytophone on varied human malaria species, our data conclusively demonstrate the ability of PAFC to detect the most important human species, *P. falciparum*. Indeed, signals generated appeared more robust than those from ex vivo murine *P. yoelii* samples. We anticipate that the PAFC will be able to detect all species, as other Hz-based devices have demonstrated, and additional study is underway to differentiate species based on the above parameters.

The rainbow (i.e., multicolor) Cytophone features multispectral laser diodes with many currently available discreet wavelengths ranging from 630 to 1650 nm as well as a time-color-coded mode^9,19,20^, which opens unique opportunities for multicolor (up to 30–40 colors simultaneously) in vivo flow cytometry of multiple targets. Indeed, although we applied this new platform to malaria in this study, the obtained data (e.g., Fig. 3, 4, 5, S3), as well as our previously published results with convention lasers^9,12,16–21,43–46,49^, indicate that our Cytophone platform with laser diodes as small as a few mm can have some potential for the label-free diagnosis of melanoma, bacteremia, sickle cell anemia (e.g., hemoglobinopathies), stroke, COVID-19-related complications (e.g., clotting), different forms of Hb including oxy-, deoxy-, and metha-Hb (methemoglobinemia)^19^ and some blood disorders associated with inadequate oxygen transport and thalassemia. Although laser diodes have lower pulse energy, on average, compared to solid lasers, the high sensitivity of the Cytophone, along with advanced software and signal processing algorithms, at least partly if not fully compensates for this limitation. Additionally, progress in developing powerful laser diode arrays with pulse energy up to 1 to 2 mJ^37^ will significantly lessen the impact of this limitation for clinical applications. While in vivo data presented here are largely limited to murine malaria, our promising in vitro* P. falciparum* data have led us to initiate a first-in-human pilot study with the rainbow Cytophone. Further work will assess the ability of Cytophone to detect/distinguish *Plasmodia* from other erythrocytic pathogens such as *Babesia* and *Bartonella*, which we anticipate will be accomplishable, given neither of the latter two are known to produce hemozoin.

We have previously demonstrated that PA signals (with typical widths of 0.2–0.3 µs) have a well-distinguishable acoustic time delay (0.5–2 µs) from circulating objects in blood vessels which are captured by the transducer above the skin as compared with background PA signals from the pigmented skin layer^21^. Thus, the time-resolved detection mode in Cytophone allowed us to eliminate the influence of skin pigmentation background on the PAFC sensitivity. In other words, this mode, along with fast signal processing algorithms, make PA data tolerant to skin pigmentation. We additionally utilized both an in vivo animal (Fig. 4a) and vessel phantom model comprised of plastic tubes and skin phantoms^38^ with a melanin layer on the phantom’s surface to assess the impact of the skin layer on PAFC readings.

In conclusion, a Cytophone with multispectral, time-color-coded pulse laser diodes and high-speed, time-resolved signal processing overcomes the previous limitations of PA techniques, particularly PAFC associated with the use of solid lasers, due to the technical complexity, large size, high cost, and one wavelength used in most applications. Our preliminary results demonstrate the feasibility of a portable, potentially battery-based Cytophone with laser diodes. The Cytophone can continuously monitor, in real-time or periodically exam blood circulation to detect the appearance of the parasites within a few hours after invasion. The Cytophone depth is well within the documented capability of conventional in vivo PA imaging techniques to assess relatively deep human blood vessels (up to 3–5 cm)^2–9^. However, these techniques using microscopy and tomography platforms are relatively slow, making it difficult to detect fast-moving (3–10 cm/s even in 1 mm depth vessels), circulating iRBCs, and other targets, especially in deep, large vessels. Our previous data with high pulse repetition solid lasers and now with laser diodes suggest that the PAFC-based Cytophone platform with acoustic resolution can provide high-speed detection and spectral identification of circulating objects of different origins in relatively deep (1–2 cm) and large (4–6 mm) blood vessels with flow velocities up to 10–20 cm/s^9,12,21^. The unique ability to interrogate such vessels while targeting the process of Hz formation, central to all *Plasmodia* species, offers the promise for very rapid, noninvasive, label-free pan-species malaria diagnosis. Taking advantage of the ability to screen larger volumes of circulating blood, Cytophone sensitivity may additionally allow for the detection of the large reservoir of asymptomatic individuals, an advancement that could help push towards both improved malaria control and its eventual elimination. Moreover, coupling data from this study with our previous PAFC work, we anticipate that the multicolor Cytophone platform may allow for the detection and identification of multiple diseases, making it a promising portable non-invasive tool in a wide range of global settings.

## Methods

### General Cytophone schematic

The multicolor PAFC-based Cytophone setup (Fig. 1a, 2a–c) is equipped with two pulsed laser diodes (MM25004, MM25005, Quantel Laser, France) with wavelengths of 808 nm and 915 nm (Fig. 2c), pulse widths of 27 ns, pulse repetition rate of 1 to 3 kHz and pulse energies of 140 μJ and 110 μJ, respectively. Each laser has 20 emitters with a total length of 5 mm and an individual emitter size of 8 × 200 µm. Both laser diodes had output divergence angles of 40 degrees for so called the “fast axis” and 10 degrees for the “slow axis” without additional optical components^37,38^ .^.^ To decrease the divergence angle in the fast axis direction to about 2 degrees and thereby collimate the laser radiation, fast axis collimator (FAC) lenses were added, each with a focal length of 260 μm and diameter of 400 μm. Two mirrors were utilized to combine the laser radiations into the same propagation direction. The one mirror was silver (PF10-03-P01, Thorlabs Inc) to reflect 808 nm diode laser radiation to eventually coincide with the 915 nm laser diode using dichroic mirror (Di02-R830-25 × 36, Semrock, IDEX Health & Science, LLC). The combined laser beams were further transformed in the focused linear beam by a spherical lens (LA1274-B, focal length of 40 mm, Thorlabs Inc.) followed by an aspherical cylindrical condenser (ACL2520-B, focal length of 20 mm, Thorlabs Inc.). The final laser beam size was 1.8 mm × 65 μm controlled periodically with a CMOS camera (DCC1645C-HQ, Thorlabs, Inc.). The pulse energies after exiting the optical system were adjusted to 80 μJ, which was adequate for our application. The laser energy was controlled using an optical power meter PM100USB, S314C sensor (Thorlabs, Inc.).

Laser-induced acoustic waves in the samples were detected with a custom-made focused cylindrical transducer (central frequency: 51.1 MHz, focal distance 6 mm, lateral acoustic resolution of 65 μm). The transducer was fixed on an independent XYZ-positioning stage to allow micrometer-precision adjustment of its position. The independent Z-stage was used to adjust the optical lens in the Z direction to maximize PA signal amplitude. To obtain equal PA signals from both laser diodes, we first balanced the energies for both laser diodes and then used the independent Z-stage to adjust the sample at optimal points for the acoustic and optical focuses by moving the stage in the Z direction. As a result, equal maximal PA signals from objects with similar absorption (e.g., black tape) on both laser diode’s wavelengths were achieved. Conventional ultrasound gel was employed for acoustic coupling. A standard pulse generator was utilized to generate a signal to drive the laser diodes, and a resulting synchronized signal was sent to the data acquisition board (ATS9350, Alazar Technologies, Inc., Canada). The PA signals were amplified by a preamplifier (AH-2010–100, Precision Acoustics Ltd, United Kingdom).

### PAFFC platform

The principles of PAFFC integrating PAFC and FFC modules have been described in detail elsewhere^14^. Briefly, the setup was built on a Nikon Eclipse E400 (Nikon Instruments Inc., USA) microscope platform with three high pulse repetition rate (1–10 kHz) nanosecond (0.6–8 ns) lasers with the corresponding wavelengths of 532 nm, 671 nm, 820 nm, 1060 nm, and 1064 nm for PA detection and with a CW488 nm laser diode for the fluorescence detection. We used a Yb-fiber laser YLPM-0.3-A1-60–18 (IPG Photonics Corp.) operating at a wavelength of 1,060 nm, a pulse rate of 1 to 10 kHz, and a pulse width of 0.8 to 10 ns. Laser energy was controlled with a power meter (PM100USB, S314C head, Thorlabs, Inc.). The signals from the photodetector (PDA10A, 150 MHz, Thorlabs, Inc.) triggered the data acquisition hardware. To form the laser beam into a narrow line (6.5 μm × 780 μm) crossing the phantom or real blood vessel in an animal model, we used a combination of aspheric (C560TME-C) and cylindrical (LJ1598-L1-C) lenses (Thorlabs, Inc.).

Laser-induced acoustic waves were detected by custom cylindrically-focused ultrasound transducers with a 28-µm polyvinylidene fluoride pressure-sensitive element with a broadband frequency response in the range of 0.2 to 32 MHz, and a focal distance of 6 mm. The transducer was mounted on an XYZ-stage to allow precise position adjustment. A PC was utilized to record transducer signals, which were amplified by an amplifier (model 5662B, 50 kHz—4 MHz, Panametrics). An analog-to-digital converter board, as well as *LabVIEW* and *MATLAB* software, enabled the setup to collect the signals. Fluorescence signals from the photomultiplier tube were sampled at a rate of 4 MHz and downsampled to 10 kHz, with an average of 400 points. Customized software was used to analyze both signals, which were shown as signal traces with the time of appearance, amplitudes, shapes, and widths for each peak higher than the background level.

### In vitro PT and standard microscopy

The general principle of the PTM system has been described in detail elsewhere^34–36^. Briefly, the technical platform of collinear dual-beam (pump-probe) confocal PTM imaging was based on an IX73 microscope platform (Olympus America, Inc., Central Valley, PA) with different high-pulse-rate (1–10 kHz), nanosecond (5–10 ns) lasers with fixed wavelengths (532/671/820/1064 nm) that used a combination of lens and fiber-optics to deliver laser lights to samples. This was achieved using a 3-wavelength Wavelength Division Multiplexer (WDM-RGB46HF, Thorlabs, Newton, NJ) that combined the beams from a 473 nm CW laser (for fluorescence excitation), 532 nm CW and pulse lasers (for PT pumping and fluorescence excitation), and 635 nm CW laser (PT probe) into a single-mode fiber. Depending on the application, this schematic allows the user to switch between pulsed and CW pump laser sources for PT imaging without compromising system alignment. First, we used conventional pumping schematics with a CW laser operating at 532 nm. Laser intensity was modulated using an electro-optical modulator (EOM-NR-C4, Thorlabs, Newton, NJ) at a frequency of 100 kHz. PT phenomena resulting from the absorption of pumping laser light were detected through modulation of probe beam intensity using a photodiode with a lock-in amplifier (SR530, Stanford Research Systems, Sunnyvale, CA). Secondly, a 532 nm pulsed laser (Model, LUCE 532; pulse width, 5 ns; pulse rate, 10–30 kHz; Bright Solutions, Italy) was used to induce linear and nonlinear PT effects in the sample. The intensity of this laser was modulated via the same electro-optical modulator to quickly change pulse energy between pulses using PT signals as feedback to prevent sample damage. To provide a confocal PT configuration, a photodetector pinhole was positioned on a plane situated one Rayleigh range away from the “waist plane” of the probe beam. Thus, the pinhole allows the detection of highly localized thermal lens effects within individual absorbing targets while eliminating the influence of out-of-focus PT signals. For 3-D imaging, successive PT images were acquired on parallel *x*–*y* planes distributed along the z-axis. To validate PT data and make the system more universal, we integrated PTM with PA, transmission, and fluorescence methods by using confocal PTM with confocal fluorescence microscope modules and the same pinhole for spatial selection of fluorescence and probe beams as well as multimodal PT-fluorescence contrast agents. In particular, the fluorescent module was used for acquiring cell auto-fluorescence and laser-induced fluorescence from cytoplasm and membrane *P. yoelii 17X* GFP + infected RBCs (using 575/15 nm channel with 532 nm excitation laser).

### Scanning electron microscopy and transmission electron microscopy

A JSM-7000F SEM (JEOL USA, Peabody, MA) with a field emission electron gun was used in this study, equipped with an EDS system (EDAX Inc, Mahwah, NJ). For SEM imaging, two sample preparation methods were utilized: 1) dry powder of Hz crystals were suspended in ethanol solution. A few drops of the suspension were deposited onto a 12.2 (diameter) × 10-mm (thickness) aluminum disk (Ted Pella, Inc, Redding, CA), and 2) the same dry powder was sprinkled on a holey carbon-coated TEM grid (SPI Supplies, West Chester, PA). TEM images were collected by a JEM-2100F with a field emission electron gun (JEOL USA, Peabody, MA), which was also equipped with an EDAX EDS system. Hz crystals were suspended in ethanol and dispersed carefully by gentle shaking. A few drops of Hz suspension were deposited on holey carbon-coated 200 mesh copper TEM grids (SPI Supplies, West Chester, PA). These grids were dried for an hour before TEM analysis.

### Animal and human studies

Animals were used in accordance with a protocol approved by the University of Arkansas for Medical Sciences (UAMS) Institutional Animal Care and Use Committee. The female C57BL/6 and Foxn1(nu/nu) mice (The Jackson Laboratory, Inc.) were infected with 10^5^ blood stage *Plasmodium yoelii* 17XNL GFP^+^ parasitized RBCs via intraperitoneal injection. Uninfected C57BL/6 mice were used as negative controls. During experiments, the mice were anesthetized by inhalation of 1.2% isoflurane and placed on their back on a heated stage at 37 °C for in vivo monitoring. Vessels chosen for the study were approximately 50 to 70 µm in diameter at a depth of 150 to 200 μm in the mouse ear. The transducers and optical tips were placed gently above the skin at the point of detection and adjusted in real-time by optimizing alignments for maximum and stable PA signals. Ultrasound gel (Aquasonic Clear, Parker Labs, Inc.) was used for acoustic coupling between the transducer and skin. Mouse blood was collected from tail vessels for in vitro testing using Giemsa staining of thin blood smears and fluorescence microscopy. For PAFFC (above), we collected blood from the ventral artery and lateral vein of the tail in anesthetized mice using a 28G needle and 1 cc syringe with sodium citrate as an anticoagulant. Blood smears were prepared and then examined on glass slides by using blood samples collected from healthy and infected mice, fixed with 100% methanol, and air dried. The mice were infected with the GFP-expressing parasites, while the uninfected mice were served as control. PA signal number per second was analyzed to estimate the blood parasite stage.

In selected experiments, human blood from volunteers was used in accordance with protocols approved by the Yale University Institutional Review Board. Informed consent was obtained from all subjects. All methods were performed in accordance with the relevant guidelines and regulations. The study was carried out in compliance with the ARRIVE guidelines.

### Dynamic vessel-flow-cell phantoms

In vitro PA detection of flowing light-absorbing objects was performed on an in-house constructed dynamic blood flow-vessel phantom^38,39^. This consisted of polyvinyl chloride plastisol with TiO_2_ nanoparticles modeling biotissue with adequate scattering and absorbing properties, a melanin layer representing pigmented skin, glass and plastic tubes with different diameters and depths, and a flow module to pump moving objects with velocities between 0.5 and 5 cm/s. In particular, a cylindrical glass tube with a diameter of 0.78 mm (Sutter Instrument, BF100-78–10) acting as a blood vessel with a depth of 0.5 to 2 mm modeled PAFC’s typical medical application. To mimic flowing cells and particles, we used 1) magnetic beads with different sizes and specific absorption in the NIR range (Fig. 1c); 2) red CBCs representing RBC aggregates in blood; 3) 100 µm transparent silica beads as white CBCs phantoms; 4 ICG aggregates absorbing preferentially at 808 nm; and 5) commercially available Hz. Specifically, synthetic Hz suspension in PBS solution at concentrations of 10 µg/mL, 1.0 µg/mL, and 0.1 µg/mL was used as the phantom of pigment skin and iRBCs. The Hz suspension in PBS was derived from a stock suspension of 5 mg/mL by adding 1 mL of PBS to 5 mg of Hz crystals in its original vial as directed by the manufacturer (Invivo Gen). To calibrate the PAFC system, the trace was also monitored after administration of magnetic beads (Spherotech Inc.) with different mean diameters. The same volume (2 µL) of magnetic beads suspension (1% w/v of different mean particle size distributions: 5.25 µm, 8.22 µm, and 23.7 µm) was suspended in 8 mL of PBS.

### Plasmodium parasite species

*Murine studies*. Using the PAFFC platform, we attempted early detection of native Hz ex vivo with the non-lethal rodent *Plasmodium* species *P. yoelii* 17XNL (MR4, BEI Resources Repository). To provide secondary verification of ex vivo real-time Hz detection, a green fluorescent protein (GFP)-tagged parasite line (*P. yoelii* 17XNL:PyGFP) was used, enabling the utilization of a fluorescent channel in the PAFFC platform. Infected blood from a Foxn1(nu/nu) mouse obtained 3 to 4 h post-infection was analyzed concurrently with the PAFFC procedure and by microscopic thin blood smear slide examination. Specifically, 100 µL of a GFP^+^ *P. yoelli-*infected blood sample was diluted in 100 µL of RPMI 1640 medium in a heparin tube. A 1 µL sample of this solution was further serially diluted in PBS in the following ratios 1:10^2^, 1:10^3^, 1:10^4^, and 1:10^5^, and each diluted sample was analyzed by PAFFC with a pulsed laser at 671 nm for PA excitation and a CW laser at 473 nm for fluorescence excitation.

#### Plasmodium falciparum studies

 Frozen samples of the *P. falciparum* (laboratory strain 3D7) were thawed using a multi-step saline procedure (BEI Resources, Manassas, VA). Plates of *P. falciparum* were maintained in continuous culture at 2% hematocrit with human erythrocytes and were incubated at 37 °C in supplemented RPMI 1640 medium under an atmosphere with 5% carbon dioxide and 1% oxygen. Once the parasite cultures reached > 5% parasitemia, they were synchronized with a 5% sorbitol solution to include only 0 to 12-h post-infection ring-stage parasites. These culture plates were monitored until parasites reached the late schizont stage, at which point a 60% Percoll gradient was used to separate the schizont-stage parasites. The collected schizont parasites were reintroduced to a new culture plate with uninfected human erythrocytes and allowed to incubate for 4 h. These *P. falciparum* cultures were synchronized with a 5% sorbitol solution to create plates containing only 0 to 4-h post-infection ring-stage parasites. The synchronized *P. falciparum* cultures were monitored for the next 48 h with a serial sampling of the culture every 6 h to prepare thin and thick blood smear microscopy slides for analysis. Nine total samples were taken of the synchronized cultures, including the following time points after erythrocyte infection: 0–4 h, 6–10 h, 12–16 h, 18–22 h, 24–28 h, 30–34 h, 36–40 h, 42–46 h, and 48–52 h.

### Data processing and statistical analysis

Mice were monitored in vivo every 1 to 3 days from 10 min to 1 h, depending on the parasitemia stage. PA signal rate was calculated as the number of signals per time unit (second or minute). To equalize the data variations due to vessel size, position of day-to-day monitoring, as well as mouse individuality, similar vessels with a diameter of ~ 50 µm in the mouse ear were selected and used. Peak-to-peak amplitudes of PA waveforms were then traced, and the points at least 3σ above the background levels were considered as the PA signals. All measurements were performed at least three times, and the averages for all these data points were plotted in the figures. Collected data (*M* counts) were plotted as *M* ± *SD*. Signal and statistical analyses were performed in MATLAB (MathWorks, Inc.). Averaging of collected data with standard error bars was calculated, and α = 0.05 significance level was employed for comparison.

## Supplementary Information

Supplementary Information 1.

Supplementary Video S1.

## Data Availability

All data associated with this study are present in the paper or the Supplementary Materials.
