# Supplementary material for: Towards rainbow portable Cytophone with laser diodes for global disease diagnostics
Source: Sci Rep. 2022 May 23;12:8671. doi: 10.1038/s41598-022-11452-w (PMC9126638; doi:10.1038/s41598-022-11452-w)
Supplement: Supplementary file 1 — Supplementary Information 1. [file 41598_2022_11452_MOESM1_ESM.docx]

**Supplementary Figures**


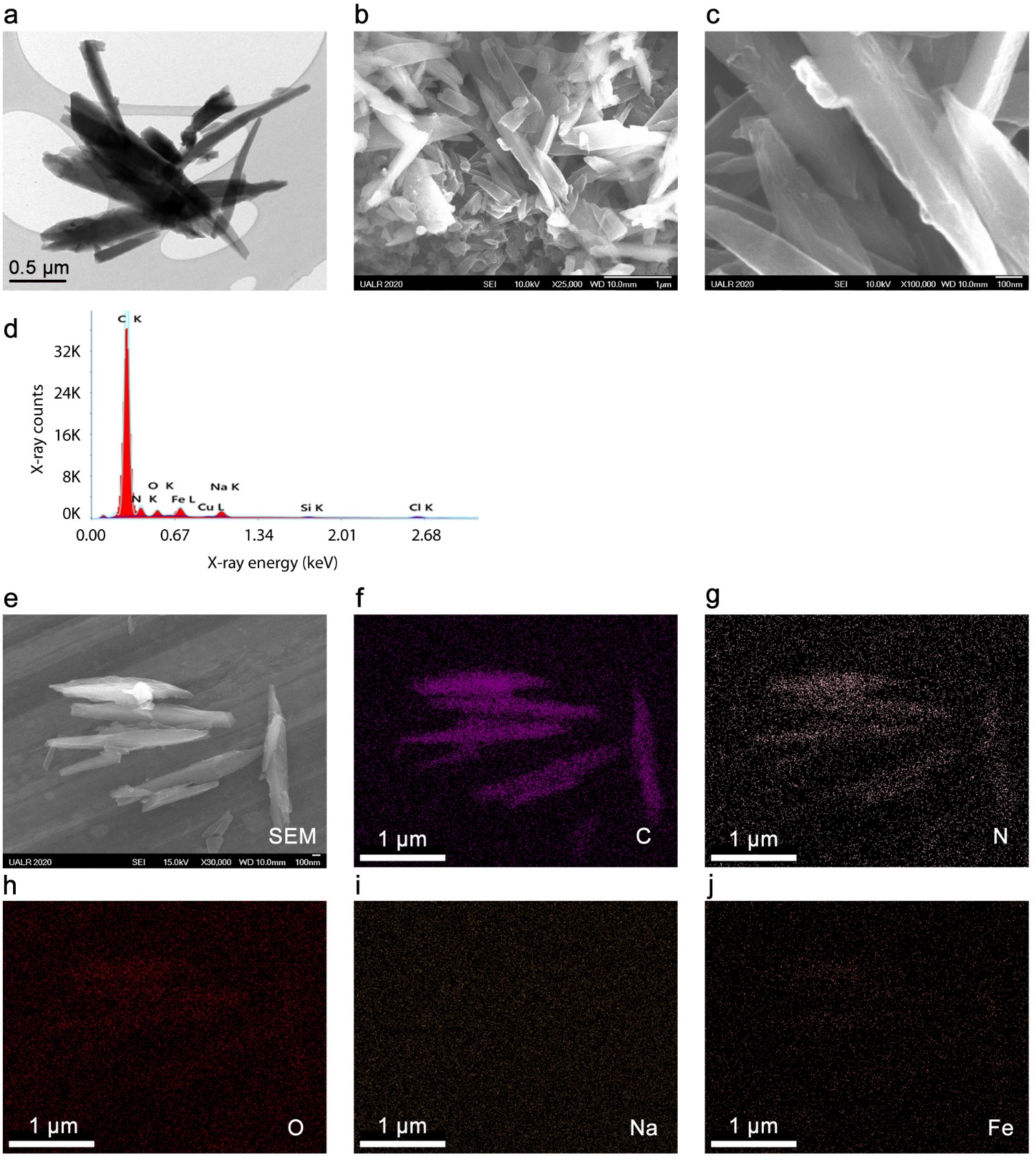


**Fig. S1 |** (**a**) TEM image of a small cluster of Hz nanocrystals. (**b**) Low-magnification SEM image of Hz nanocrystals. (**c**) High-magnification SEM image of (**b**). (**d**) EDS taken from (**b**). Even though carbon is the most prominent component of Hz, the C peak in this spectrum is slightly enhanced due to the carbon film support (~3 nm) used as a sample holder for the Hz nanocrystal powder. Aside from **c,** the other elements present on this spectrum are N, O, Fe, and Na. (**e**) SEM image of a few Hz nanocrystals. EDS elemental map of carbon (**f**), nitrogen (**g**), oxygen, (**h**), sodium (**i**), and iron (**j**).

**
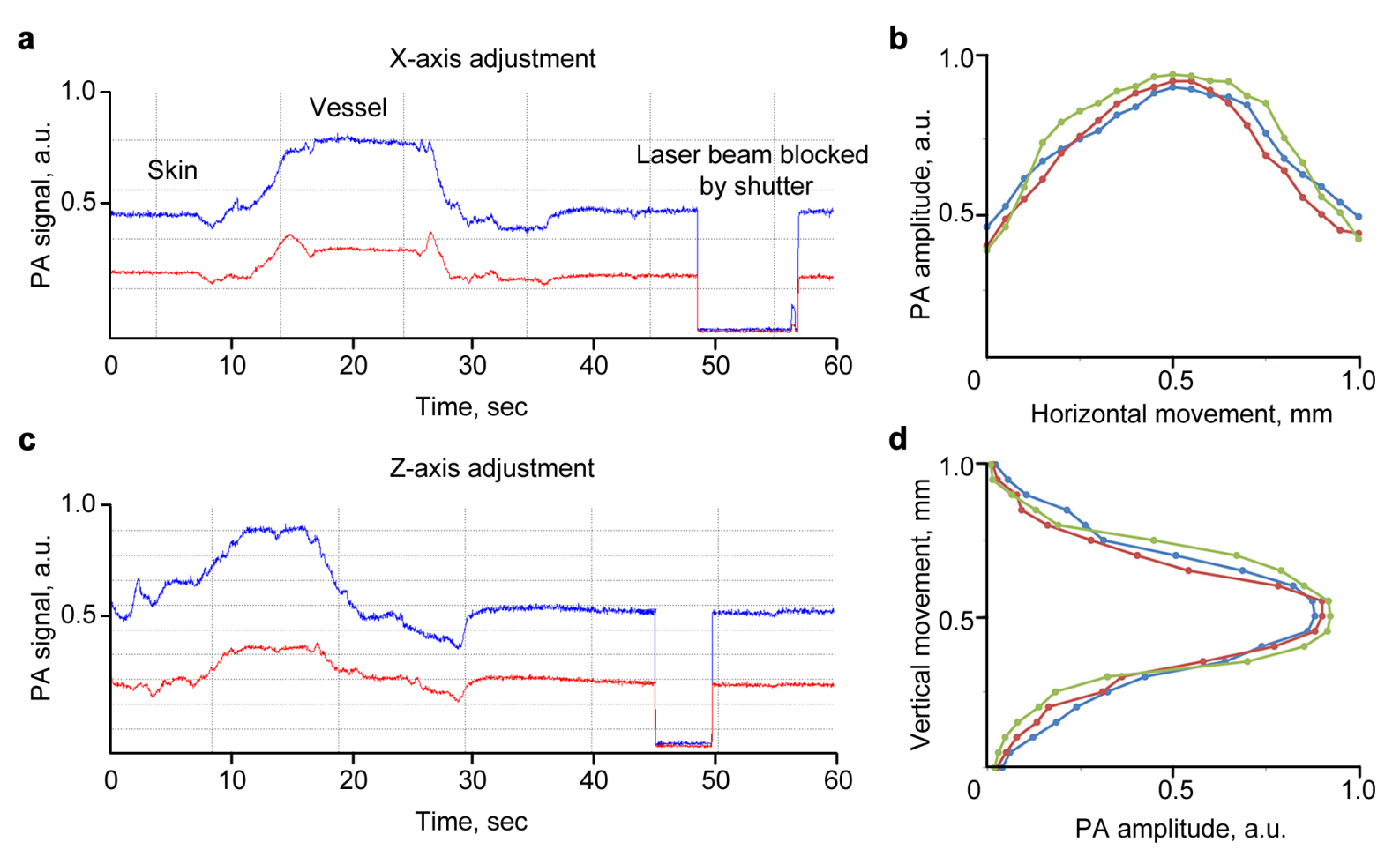
**

**Fig. S2 |** PA navigation of linear laser beams with 808 nm (blue) and 915 nm (red) on blood vessels in mouse model. (**a**,**b**) Comparison of PA signals from a vessel and surrounding skin in mouse model during laser scanning across the vessels. (**c**,**d**) Maximization of PA signal amplitude from a vessel during “horizontal“ (lateral) and “vertical” (axial) laser beam scanning at 808 nm three times in direction forward (green), backward (blue) and forward again (red).


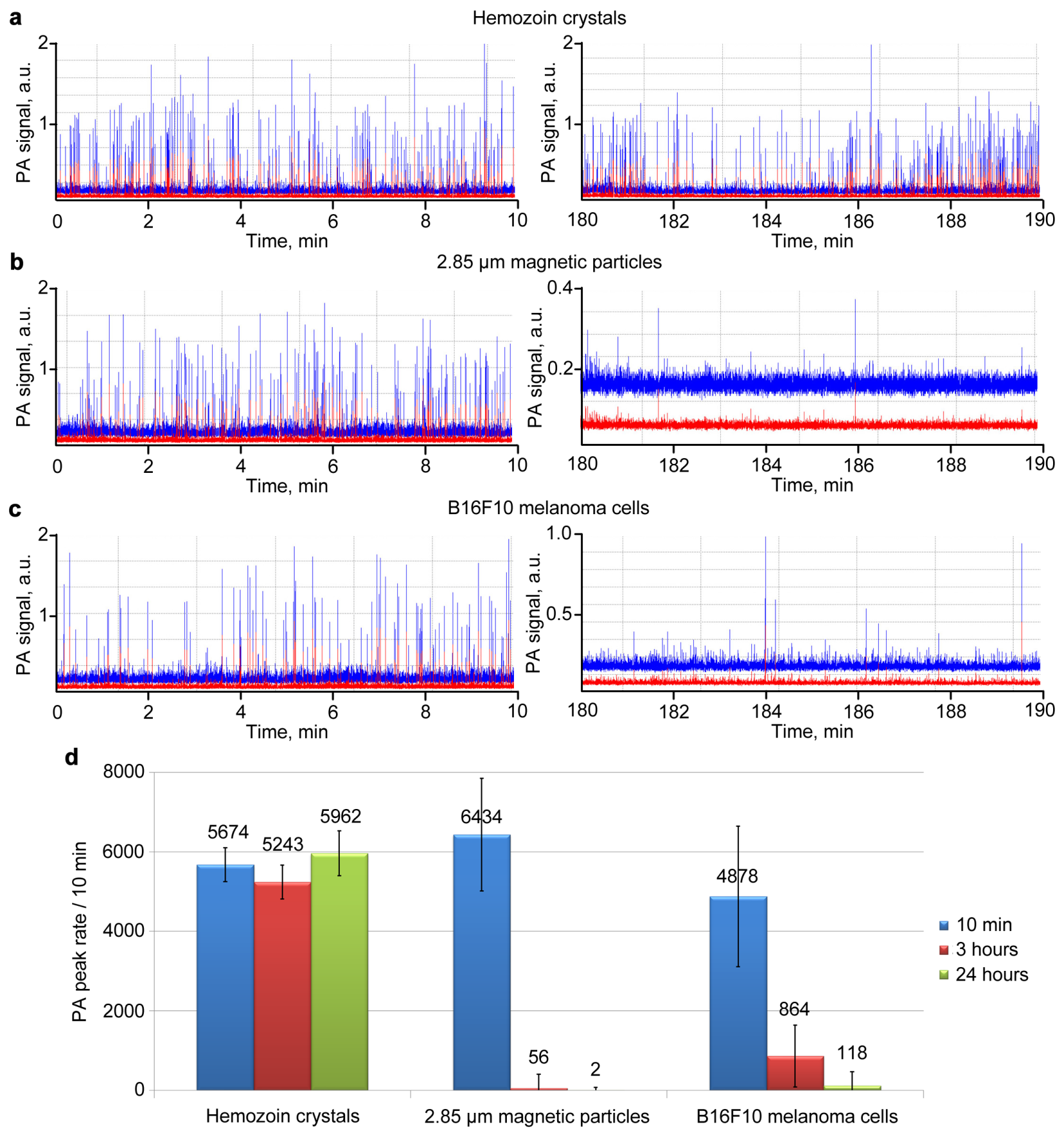


**Fig. S3 |** *in vivo* PA monitoring of circulating agents in mouse ear vessel immediately (left) and after 3 h (right) their injection in tail vein at 808 nm (blue) and 915 nm (red). (**a**) Commercially available Hz. (**b**) 2.85-µm in diameter of magnetic particles. (**c**) B16F10 melanoma cells. (**d**) PA peak rate (number of PA signals per 10 min) 10 min, 3 h, and 24 h after injection of Hz, magnetic particles, and melanoma cells in mouse circulation.


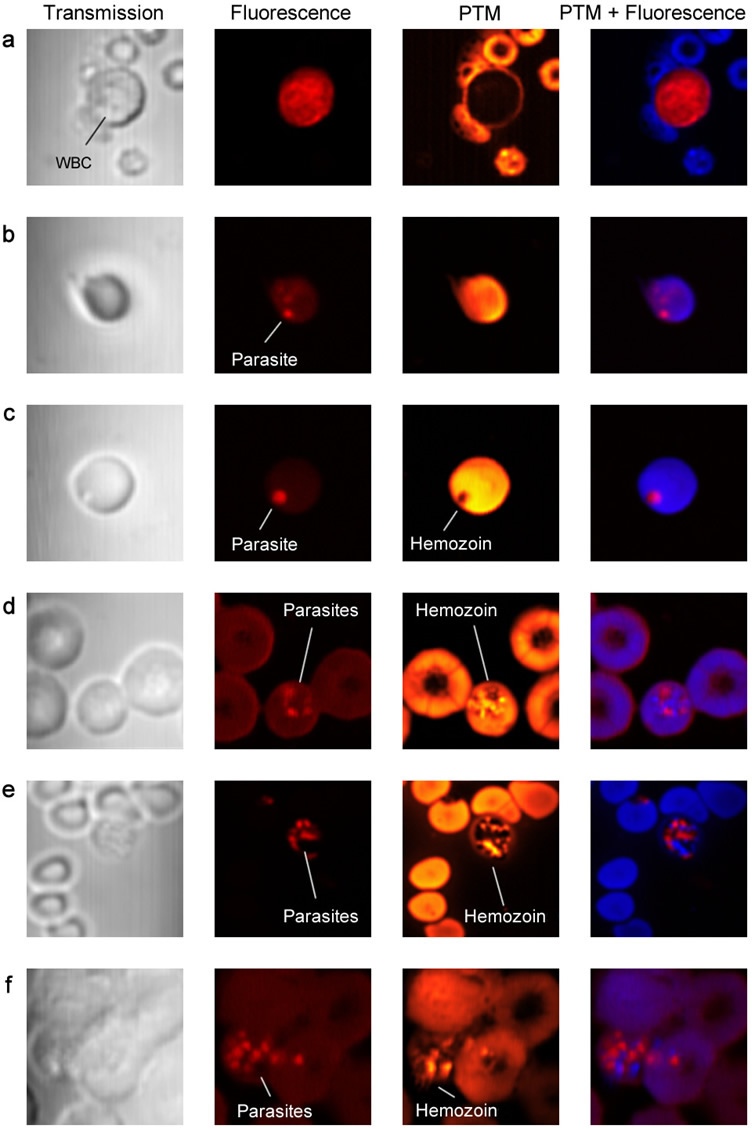


**Fig. S4 |** Different types of optical images of individual control and *P. yoelii* infected with cells stained with propidium iodide (PI). Columns: Transmission (**first**), fluorescent (**second**), PTM (**third**) and combined (**fourth**) images of mouse blood cells. (**a**) control sample with RBCs and single WBC with no signs of parasites or Hz. (**b**) Early ring stage (2-4 hours after invasion) with single parasite and no Hz yet. (**c**) Early ring stage (3-4 hours after invasion) with single parasite and single Hz. (**d**) Trophozoite stage (4-8 hours after invasion) with 2 parasites and few Hz. (**e,f**) Schizont stages (~24 hours after invasion) with many parasites and Hz.


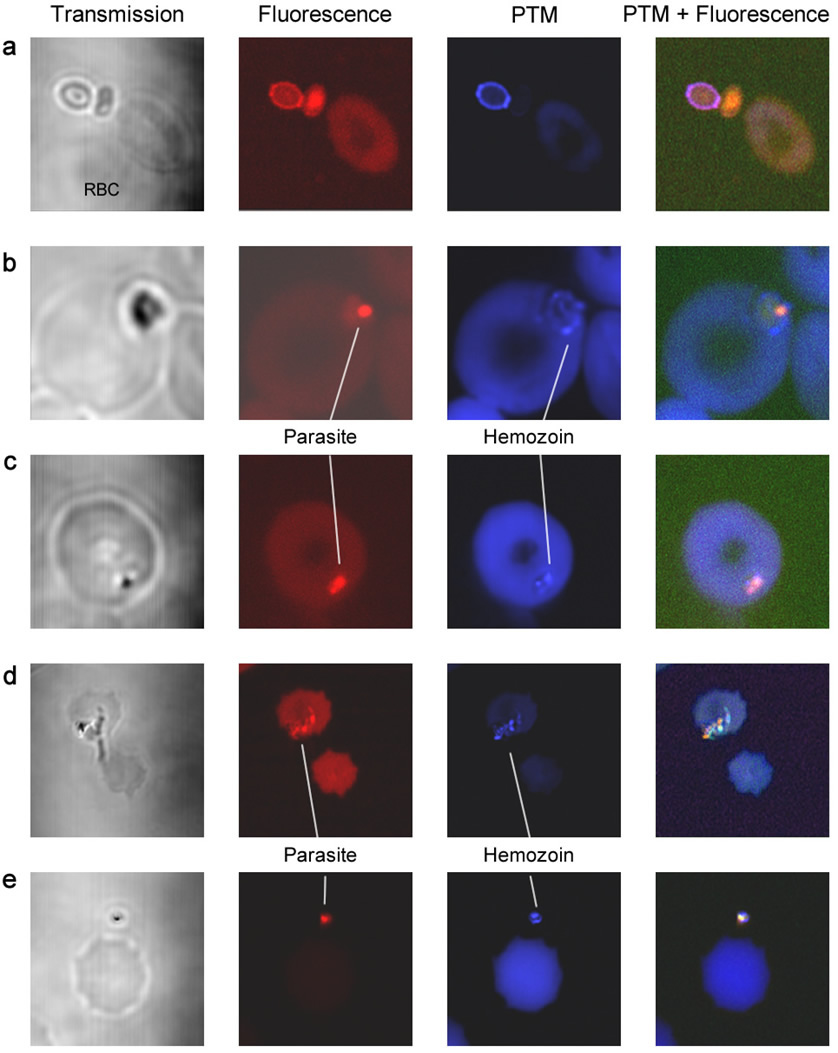


**Fig. S5 |** Different types of optical images of individual control and *P. faciparum*- infected human cells stained with propidium iodide. Columns: Transmission (**first**), fluorescent (**second**), PTM (**third**) and integrated (**fourth**) images of human blood cells. (**a**) Early stage (0-4 hours after parasite delivery to RBCs suspension) with the parasites outside RBCs before invasion. (**b, c**) Early stage (2-4 hours after infection) with single parasite in RBC with one (**c**) and a few (**b**) Hz. (**d**) Matured stage (24 hours after infection) with many parasites and Hz. (**e**) Extracellular parasite associated with Hz.


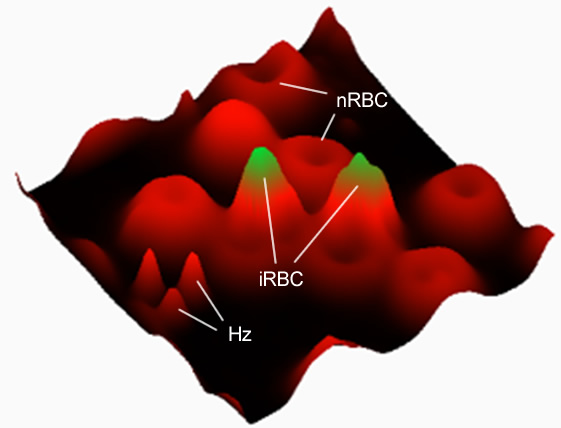


**Fig. S6 |** 3-D simulation of PT image of mixed iRBC and nRBCs.


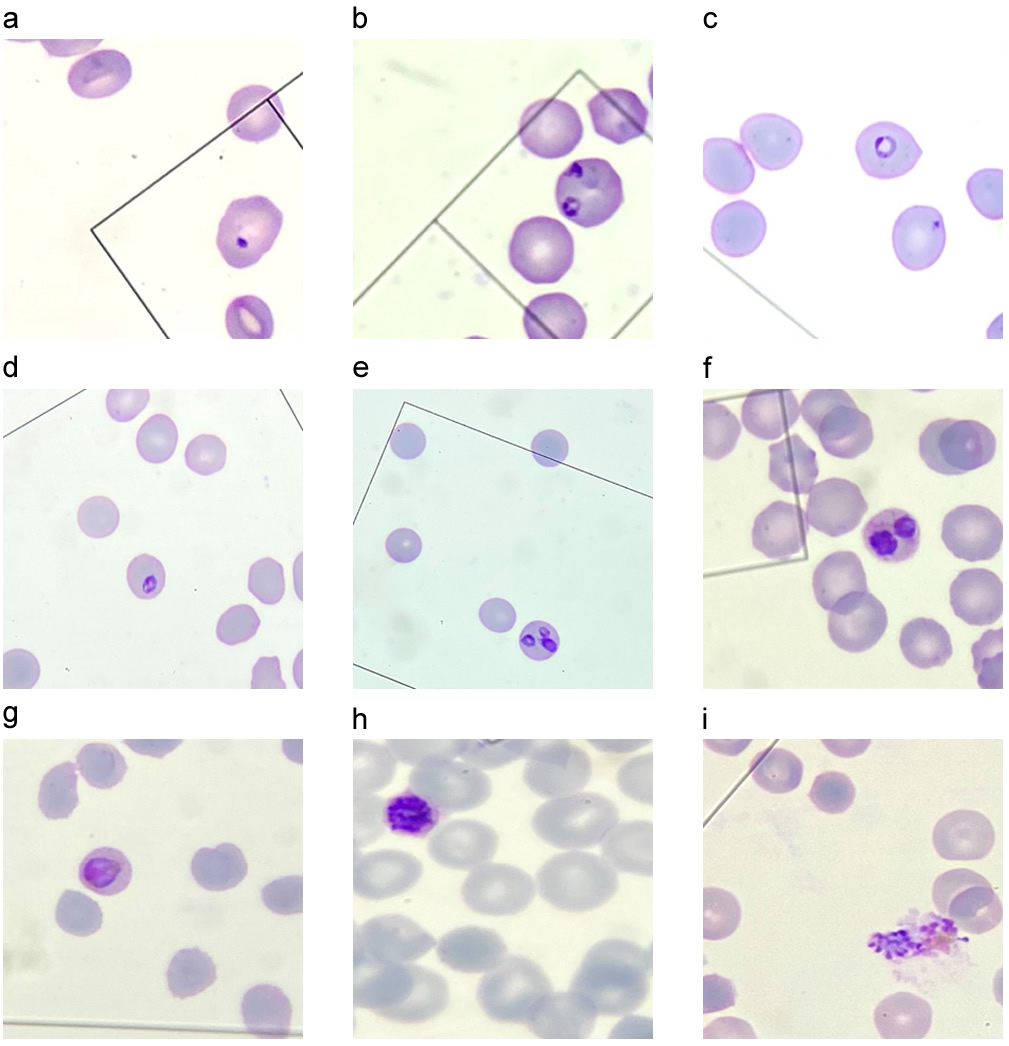


**Fig. S7 |** Smear microscopy images of iRBCs at different 9 time point post-infection of RBC with *P. falciparum*: (**a**) 0-4h, (**b**) 6-10h, (**c**) 12-16h, (**d**) 18-22h, (**e**) 24-28h, (**f**) 30-34h, (**g**) 36-40h, (**h**) 42-46h, (**i**) 48-52h.

**Supplementary Video**

Video of 3-D PT image rotation of single iRBC.
